# Suicide among transport industry workers: A systematic review and meta-analysis

**DOI:** 10.5271/sjweh.4059

**Published:** 2022-10-29

**Authors:** Sharna Mathieu, Victoria Ross, Rachmania Wardhani, Paula Brough, Darren Wishart, Xi Wen Chan, Kairi Kõlves

**Affiliations:** 1 Australian Institute for Suicide Research and Prevention & World Health Organization Collaborating Centre for Research and Training in Suicide Prevention, School of Applied Psychology, Griffith University, Brisbane, Australia.; 2 Centre for Work, Organisation, and Wellbeing, Griffith University, Brisbane, Australia.; 3 School of Applied Psychology, Griffith University, Brisbane, Australia.

**Keywords:** aviation, heavy vehicle, maritime, men’s health, mental health, transport worker, workplace suicide prevention

## Abstract

**Objectives:**

Working in high-stress and male-dominated occupations is associated with an elevated risk of suicide. The current study sought to conduct the first systematic literature review and meta-analysis aimed at determining suicide risk across the diverse, high pressure and male-dominated transport industry (commercial aviation, merchant seafaring, transit/driving) as compared to the general/employed population.

**Methods:**

Searches of PubMed/Medline, Scopus and PsycINFO databases were conducted without date restriction until March 2021. Studies were included if they were written in English, were peer reviewed, and presented primary observational research data. Studies referring exclusively to suicidal ideation, suicide attempts, self-harm, and/or accidents were excluded.

**Results:**

Following deletion of duplicates and non-English titles, a total of 4201 titles/abstracts were screened and 92 full-texts were read against inclusion/exclusion criteria. The final included sample consisted of 23 articles (16 used for meta-analysis). Results from the meta-analysis indicated that transport workers had a significantly elevated risk for suicide as compared to the general/employed population. Results were consistent across sensitivity analyses, and there was some variation across subgroup analyses.

**Conclusions:**

Overall, we found transport workers had a significantly higher risk for suicide than the general/employed population, and this appeared to be driven by the association for those working in merchant seafaring/maritime occupations. The findings are discussed in relation to an identified need for the development, implementation, and evaluation of tailored workplace suicide prevention strategies for transport industry workers.

Numerous interrelated and co-occurring factors at the individual, family, and social levels (1) contribute to the loss of over 700 000 lives globally to suicide each year (2). Within this complicated milieu, work-related factors such as working in situations of prolonged physical, environmental, financial, and/or psychological stress, have been associated with an increased risk of suicide (3, 4). Suicide-related occupational stressors also include workplace access and familiarity with means of suicide, such as firearms (5). These factors have been implicated in several industries at high-risk for suicide such as healthcare, correctional and defence services, construction, and agriculture (6).

One industry with high levels of inherent workplace stressors is the transport industry which is focused on the transport of people and freight by air, sea, machine/mobile plant, road, or rail. Within the transport industry, workplace stressors include physical and environmental (eg, aircraft/machine noise, sitting for extended periods of time, jetlag, motion sickness, adverse temperature), social (eg, working away from family/home, isolated during work hours), and psychological (eg, highly demanding, monotonous, strict time pressures, huge responsibilities for their own, passenger, cargo, and community safety) (4). The situation intensified during the COVID-19 pandemic where commercial aviation all but ceased creating job and financial insecurity (7, 8); passenger- or customer-facing workers risked exposure to the virus (eg, bus drivers, delivery drivers); and maritime workers risked working in close confines with infected individuals (9). The mounting strain as a result of COVID-19 occurred while demand for online retail and supply chain pressure for freight and deliveries increased dramatically (10).

Unsurprisingly, transport workers commonly report problems with sleep and loneliness (11, 12), reduced work performance and work-related safety issues (13, 14), poor mental health and wellbeing (15, 16), and are more likely to require work-related compensation for a psychological injury compared to other workers (17). Furthermore, transport workers have been shown to have higher rates of suicide than the general and employed population (18–21). A previous review (21) examined suicide risk across broad occupational groups/skill levels and found that machine operators/assemblers had the second highest risk for suicide after elementary professions (eg, cleaners). However, while this review included seafarers and transit/driving professionals in an overarching category, it also included stationary plant, machine assemblers, miners, communications, and public utilities professionals. It did not include workers from other important and high-stress transport industries (21).

To our knowledge, there has been no systematic review or meta-analysis specifically investigating suicide across the transport industry (ie, aviation, maritime, and transit/driving/mobile plant operation). The lack of a thorough review limits theoretical advancements in this area, producing knowledge gaps and potentially flawed practical recommendations. A clearer understanding of the risk for suicide among transport workers will facilitate the development and implementation of tailored workplace-based suicide prevention initiatives that are sensitive to the unique needs of this industry. Therefore, the current aim was to conduct a systematic literature review and meta-analysis to determine the risk of suicide in this industry compared to the employed and general population. We also aimed to determine whether the risk of suicide differed according to occupational or demographic characteristics of workers within the industry. Our research question was thus: Among workers, what is the effect of working in the transport industry (aviation, maritime, and transit/driving/mobile plant operation) on risk of suicide as compared to the general or employed populations?

## Methods

This review followed the updated Preferred Reporting Items for Systematic Reviews and Meta-Analyses (PRISMA) Guidelines (22) (supplementary material https://sjweh.fi/article/4059, table S1) and was prospectively registered with PROSPERO (CRD42021236485).

### Search strategy

Searches of PubMed/Medline, Scopus and PsycINFO were conducted for publications without date restriction until 4 March 2021. Search terms related to rail/railway were excluded to minimize interference from papers referring to suicides where rail/railways were the primary method of community suicides (ie, not related to direct employment in the industry). Search terms relating to skill level or social class were also excluded, as the transport industry is incredibly diverse in terms of minimal educational and skill attainment required for certain occupations. Database specific Boolean operators (eg, AND, OR, NOT) and truncation symbols (eg, *, “ “) were used. The search terms used were: suicide AND driver OR pilot OR truck OR train OR occupation* OR aviation OR maritime OR “transport worker” OR “transport industry” NOT “pilot study” OR “pilot trial” OR “pilot program” OR training.

### Inclusion and exclusion criteria

Publications were included if they were written in English, were peer-reviewed, and presented primary observational research data (ie, not editorials, reviews, single case reports or other qualitative designs). Studies referring exclusively to suicidal ideation, suicide attempts, self-harm, other non-fatal suicidal behaviors, and/or accidents were excluded. Formal definitions of transport occupations were adopted from the 2018 standardized occupational classification system (23) to determine eligible studies. Studies where a worker’s primary role included the preparation of food (eg, catering) were excluded, unless the workers were specifically identified as ship stewards or flight attendants. Aviation professionals whose primary role was in agriculture were excluded, as were maritime workers if they were working in fishing or trawling. If occupational categories were overly broad and specific information relating to transport workers could not be obtained, these studies were also excluded.

### Data extraction and quality assessment

Duplicates and non-English titles were deleted, and two reviewers independently screened the titles and abstracts against inclusion/exclusion criteria. A third reviewer was consulted when necessary to resolve any discrepancies. The full-text of the remaining articles was independently assessed against the inclusion/exclusion criteria. Prior to finalization, reference lists of included articles were screened to capture any missing papers not identified in earlier searches. Two reviewers independently extracted data from the included articles into standardized tables (using Microsoft Excel). This included author(s), transport sector, publication year, country and time of data collection, study design, rates of suicide, effect measures and 95% confidence intervals (CI), other important information, and major limitations. If study designs were not explicitly recorded in the articles, designs were determined by the extractors based on available information (24). If multiple levels of occupational groups were reported, the most narrowly defined was extracted.

There is currently a lack of consensus regarding validated quality assessment and risk of bias tools that are applicable to non-randomized, descriptive, and observational designs in the literature (25). Using a modified version (26) of a quality scoring measure (27), two reviewers independently evaluated all included studies, and the scores were discussed for consensus. This is a generic quality scoring measure for quantitative studies that was suited to the variety of study designs identified in the included articles, and which allowed for comparisons of quality across studies and subgroup analyses in the meta-analysis.

### Data synthesis and meta-analysis

Three studies utilized the same study population from Australia (5, 20, 28), three from Switzerland (19, 29, 30), and two from England and Wales (31, 32). Consequently, only the most recently published or most comprehensive study was included from each group in the meta-analysis. The effect sizes across studies included in the meta-analysis were rate ratios (RR), incidence rate ratios (IRR), and mortality rate ratios (MRR), as well as proportional (PMR) or standardized mortality ratios (SMR). Corresponding authors of six articles were contacted for additional or more specific detail (28, 32–36), but only the authors of one study (35) provided this information. Three of these articles were included in the review and meta-analyses by manually calculating RR using available results in the publication (28, 32, 36), and two of these articles were excluded as specific findings relating to suicide and/or transport workers could not be obtained/calculated (33, 34).

If a study included more than one comparison group, the employed population was preferentially extracted. If a study provided multiple effect sizes, the standardized or adjusted figures were extracted. If a study provided multiple different effect measures, RR were preferred (as well as IRR, and MRR), followed by odds ratios (OR), SMR, PMR, or comparative mortality ratios (CMR) (37). In previous occupational suicide mortality meta-analyses (21, 37), these effect measures have been combined for the purpose of meta-analysis due to the rarity of suicide in the working population (38). A pooled effect size was calculated for the risk of suicide among transport workers compared to a reference population using Review Manager (RevMan) version 5.4 (39). The I^2^ statistic was used as a measure of heterogeneity, and a random effects model was applied. A funnel plot was produced to investigate publication bias (40). Four separate sensitivity analyses were also conducted, where we removed: (i) studies that used one or only several selected occupations as a reference group; (ii) studies that were rated as low quality, (iii) studies that did not define their transport workers by standardized occupation classification systems (eg, family report, union membership), and (iv) broad categories that may have contained workers outside the transport industry.

Subgroup analyses were performed in RevMan, and a summary forest plot was produced in Microsoft Excel. These included gender (male, female, all persons), transport sector (aviation, maritime, transit/driving, general/mixed transport workers), study design, data collection period (1989 and prior, 1990–2004, and 2005 onwards), region/country, effect measure, reference group, adjustment, and study quality. If a data collection period spanned the subgroup categories, articles were included in the period when data collection finished. If a study provided effect sizes for men, women, and all persons, only the information for specific gender groups were extracted. If a study provided effect sizes for men and all persons (18), then both estimates were extracted.

## Results

**Figure 1:**
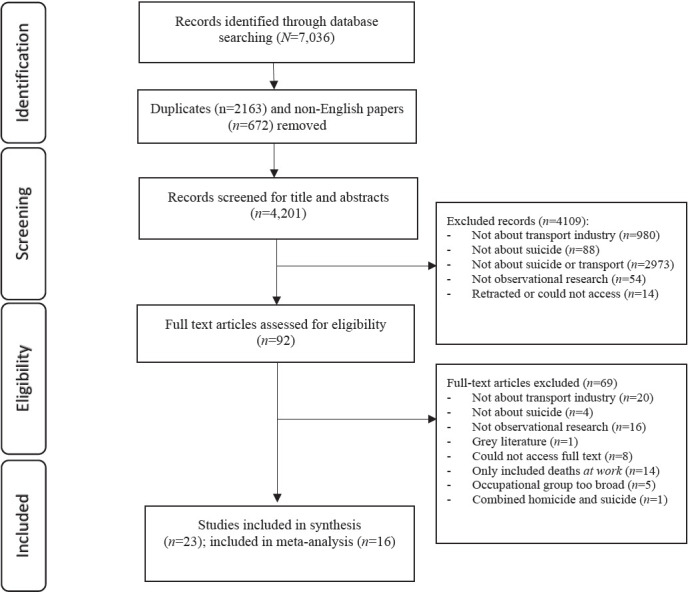
Study selection flow-diagram based on PRISMA guidelines

Searches identified 7036 articles. Duplicates and non-English titles were removed, resulting in 4201 titles and abstracts screened against inclusion/exclusion criteria. Ineligible articles were removed at this stage (N=4109), leaving 92 full-texts read against criteria. Of these, 49 articles were excluded. A further 14 articles were focused exclusively on deaths at sea/port [eg, (41)] or plane-assisted suicides [eg, (42)], highlighting a unique aspect of suicide in transport workers whereby death can occur *at their place of work*. However, these articles were subsequently excluded as they would be likely to underestimate the risk of suicide in transport workers (ie, by omitting suicides that occur outside of work or by other means in their estimates). Six additional articles were excluded as they combined suicide and homicides (34) or described broad occupational categories from which it was impossible to extract information specifically relating to transport workers [eg, (33)]. This resulted in a final sample of 23 articles, of which 16 were included in the meta-analyses (figure 1).

One study focused exclusively on transport workers in the commercial aviation sector (43), three on maritime workers in merchant seafaring (44–46), and one on bus drivers (47). These studies investigated various causes of death in a cohort of transport workers. The remaining 18 studies investigated the risk of suicide across multiple or selected occupational groups. These studies largely relied on standardized occupational classification systems from different countries of varying levels and specificity. However, three relied on other means of determining occupation (eg, loved one’s report) or it was unclear (48–50).

Regarding the types of effect measures, six studies reported RR (5, 18, 20, 30, 44, 51), two used relative risk or hazard ratios (48, 50), one used OR (52), one used PMR (47), and eight used SMR (19, 29, 31, 35, 43, 45, 46, 53). Of the remaining studies, four reported rates per 100 000 (28, 32, 49, 54) or 1 million (36), and available data was used to manually calculate RR where possible. Most included studies (N=11) were case series (5, 18, 20, 31, 32, 36, 49, 50, 52–54), followed by ten retrospective or prospective cohort studies (19, 29, 30, 35, 43–48), one case–control study (51), and one ecological design (28).

Most studies included an employed or working population as a comparison group. These included the total employed population in seven studies (5, 18, 20, 28, 35, 36, 44), four included a single low risk occupation as a reference group (30, 48, 50, 51) or, for the purpose of manual calculation, several selected high-risk occupations available in the article (32). Ten studies included the general population, often of working age, as a comparison group (19, 29, 31, 43, 45–47, 52–54). For the final study, a comparison group was difficult to determine (49).

Five of the included studies had data collection periods from 1989 and prior (44, 46–49), five from 1990-2004 (35, 43, 45, 51, 52), and twelve from 2005 onwards (5, 18–20, 28–31, 36, 50, 53, 54). One study had two distinct data collection periods, both of which were extracted, one 1989 and prior and one 2005 onwards (32).

Only two studies focused exclusively on suicide mortality among working women (30, 45), and a further three studies provided specific effect sizes for women (19, 29, 43); however, only three of these could be included in the meta-analysis due to overlapping samples (19, 43, 45). The number of suicides for women were reported in a further six studies but due to rarity were excluded from further analyses or relevant effect sizes were not calculated (5, 18, 20, 35, 36, 54). Each of these studies provided the number of suicides, and effect sizes and/or rates for men also. Information on suicide in all persons was reported in a further five studies (28, 32, 48, 51, 52). Seven studies provided information on suicide only among male workers due to a lack of female workers in the cohort or a scarcity of suicides among women in those occupations (31, 44, 46, 47, 49, 50, 53). Study summaries are provided in supplementary table S2.

### Meta-analysis: overall risk of suicide in transport workers

**Figure 2:**
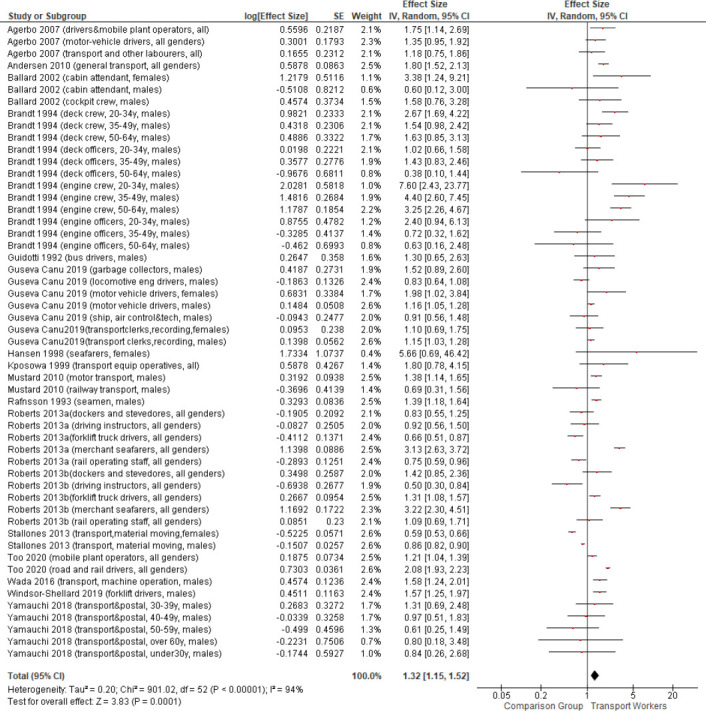
Forest plot of overall results of the meta-analysis.

**Figure 3:**
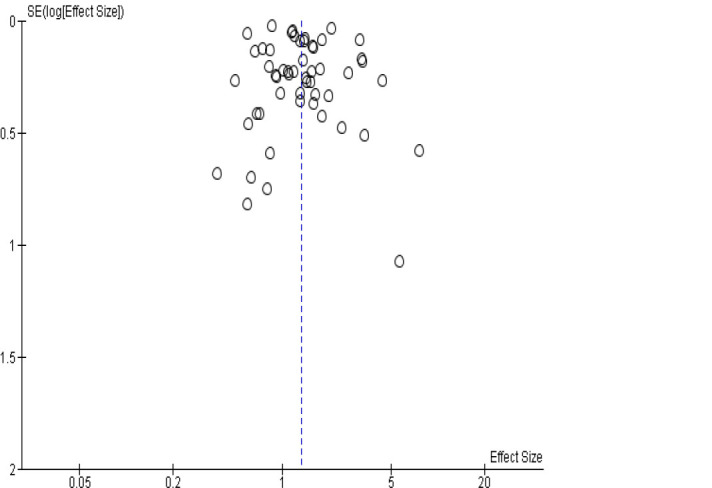
Funnel plot of included studies

Overall, based on 16 studies, transport workers had a significantly higher risk of suicide compared to the employed/general population [see figure 2; pooled effect size of 1.32, 95% CI (1.15–1.52)]. This finding was slightly stronger in the sensitivity analyses removing studies that used one or only selected occupations as reference groups [pooled effect size of 1.40, 95% CI (1.18–1.68)], removing transport categories that remained somewhat broad [eg, transport plus other laborers, material recording and transport clerks, and garbage collectors; pooled effect size 1.35, 95% CI (1.16–1.58)], and removing low quality studies [pooled effect size of 1.42, 95% CI (1.20–1.66)]. In the final sensitivity analysis, removing studies that did not rely on standardized classification systems for identifying workers, the pooled effect size reduced somewhat but remained significant [pooled effect size of 1.29, 95% CI (1.00–1.50)]. Based on visual inspection of the funnel plot (figure 3), the studies had a small standard error which were clustered symmetrically.

**Figure 4:**
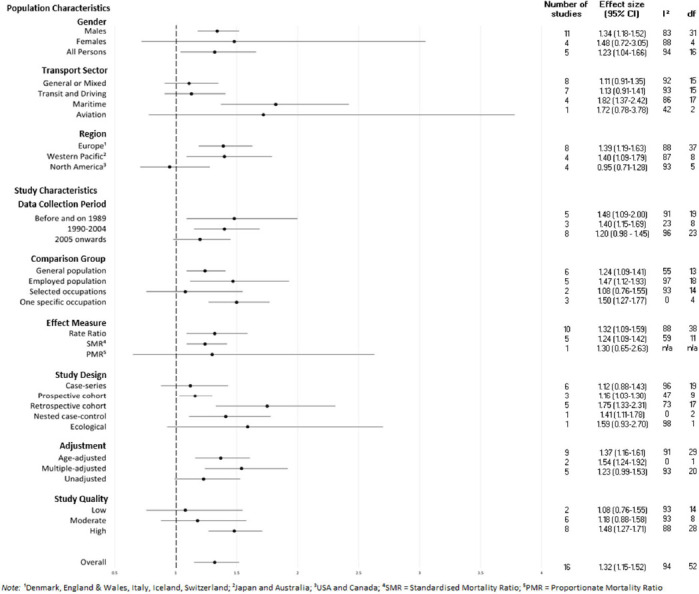
Forest plot of subgroup analyses including number of studies

All subgroup analyses are summarized in figure 4 (more detailed figures are available in the supplementary material). The test for subgroup differences was non-significant for gender (χ^2^=0.10, df=2, P=0.95, I^2^=0.0%) and region (χ^2^=5.22, df=2, P=0.07, I^2^=61.7%). For transport sector, tests for subgroup differences were significant (χ^2^=9.66, df=3, P=0.02, I^2^=69.0%). Those working in maritime/merchant seafaring occupations showed significantly greater risk for suicide than the general and employed population group whereas the remaining sub-industries did not.

The test for subgroup differences was non-significant for data collection period (χ^2^=1.84, df=2, P=0.40, I^2^=0%), comparison group (χ^2^=4.90, df=3, P=0.18, I^2^=38.8%), effect measure used (χ^2^=0.26, df=2, P=0.88, I^2^=0%), adjustment (χ^2^=2.04, df=2, P=0.36, I^2^=1.7%), and for study quality (χ^2^=3.58, df=2, P=0.17, I^2^=44.2%). For study design, the test for subgroup differences was significant (χ^2^=10.03, df=4, P=0.04, I^2^=60.1%). Studies using prospective and retrospective cohort, and nested case–control study designs produced significantly higher effect sizes of suicide rates among transport workers as compared to the comparison group, while studies using case series and ecological designs did not show significant risk of suicide.

### Quality ratings

The quality of included studies ranged from 8–19 (from a possible total of 20). Most studies were rated as ‘high’ quality (N=12), followed by ‘medium’ (N=7) and ‘low’ (N=4) quality. Descriptive and observational studies are often appropriate and widely used in determining the epidemiology of rare suicidal behaviors (24, 55). However, it is acknowledged that while these study designs are valid and can be conducted in a high-quality way, they are typically considered lower levels of evidence given a greater likelihood for risk of bias (56). It should also be noted that the two studies with the lowest quality ratings were excluded from the meta-analysis due to insufficient information. For an overview of the quality scoring see supplementary table S3.

## Discussion

This is the first review that has focused exclusively on suicide risk in this diverse and high-pressure industry. Overall, transport workers had a significantly higher risk for suicide than the general and employed populations. This finding was robust across sensitivity analyses, including removing studies where transport workers were classified in groups that may have included other laborers such as construction, which is its own high-risk industry (21). It appears that this association is driven by those working in the maritime industry (eg, deck and engine crew). Despite non-significant subgroup comparisons, the risk for suicide appears more pronounced among men and ‘all persons’ groups working in transport. Although women had a higher estimate, this did not reach significance and there were relatively fewer included studies that provided information on suicide among women in the transport industry.

Globally, suicides occur more frequently among men than women (2). The transport industry is considered a male-dominated workforce (57). Other male-dominated industries such as agriculture (37) or laborers/construction (21) have also demonstrated higher risks for suicide. It is important then to consider the impact of gender and masculine norms with regards to suicide and related behaviors in the workplace. Previous research has identified the role of masculine self-reliance and stoicism as being relevant to suicide, stigma, and help-seeking (58). These qualities have been directly linked to suicide among men working in agriculture (59). Furthermore, within a workplace context, elements of masculinity (eg, dominance, anti-weakness positioning) have also been implicated in co-creating toxic workplace cultures that favor competition and hierarchy and often lead to adverse outcomes for workers (of all genders) such as bullying/harassment, burnout, poor work–life balance, and reduced worker well-being, which has implications for the function and health of work teams and organizations [eg, (60, 61)].

These ‘masculinity contest cultures’ within organizations may also influence workers to take unnecessary risks to achieve role objectives and may help explain why certain work-related stressors impact suicide in men and women differently (60). For example, there are relatively high suicide rates among women in highly hierarchical professional occupations such as healthcare, as compared to men (62). In turn, men may have higher suicide rates than women when experiencing job insecurity or unemployment, where men are unable to prove subjective ‘worth’ and achieve social status as a ‘man’ (60). Regarding transport specifically, despite many regulations and safety practices implemented at national and organizational levels, systemic pressures to take risks such as working through fatigue, using substances, and travelling at higher speeds are well documented (63). Furthermore, organizational safety requirements and fitness for work parameters, such as preventing pilots from flying if they experience mental health difficulties, may serve to further inhibit help-seeking and contribute to ‘anti-weakness’ high stress workplace cultures. Given high rates of suicide among men working in the transport industry, and the possible impact of masculine norms and organizational values, it is essential that suicide prevention strategies and mental health promotion interventions developed for men in this industry are sensitive to, and directly accommodate, the role of masculinity and socialization of gender on help-seeking and perceived acceptability of services (64, 65). This may be especially important in the wake of the COVID-19 pandemic and associated increase in occupational and financial pressures for these workers (66).

In addition to the impact of gender and workplace stressors such as job insecurity (4), there are other organizational and individual factors that may contribute to suicide risk within the transport industry. It was not possible in the current meta-analysis to determine what impact known risk factors such as socioeconomic, relationship problems, substance use, or mental health variables had in contributing to suicide risk in transport workers. For instance, transit/driving professionals often experience a range of traumatic workplace incidents and accidents, including community suicides whereby individuals may crash into/walk in front of heavy vehicles or ‘person under train’ related events (67). Consequently, a burgeoning field of research examines mental health conditions experienced by transport workers, including acute or post-traumatic stress, and subsequent impacts on their work performance [eg, (67)]. However, a detailed exploration of how these experiences and associated mental health conditions may relate to the experience of suicidal thoughts and behaviors in these workers is lacking.

In the current review, those working in the maritime industry had an elevated risk for suicide, whereas estimates for the aviation and transit/driving sector did not reach significance. This may be a result of different levels of economic and educational attainment across industries. Indeed, certain transport workers such as commercial pilots obtain higher occupational skill levels compared to a ship’s deck crew, and previous research has shown that those working in professions with lower skill levels (including maritime and transit/driving professions) have a higher risk of suicide (21). Furthermore, merchant seafaring is a highly hazardous and isolating profession (68), associated with considerable loneliness, mental health problems, fatigue, and job strain (69–71). Importantly, loneliness combined with other factors such as self-worth, acquired capability, and perceived burden­-someness are central elements in the interpersonal theory of suicide (72).

### Implications and directions for future research

Unfortunately, in recent reviews of workplace suicide prevention (73) and men’s mental health promotion interventions (74), there were no programs specifically tailored to the transport industry. Given the elevated risk of suicide among transport workers, there is a strong need for developing, implementing, and evaluating tailored multilevel workplace suicide prevention interventions that are cognizant of interrelated workplace stressors and encourage cultural change with regards to suicide related stigma, help-seeking, and other related barriers such as less healthy aspects of masculine norms. While masculine norms are not inherently harmful, it is important that workplace suicide prevention and mental health promotion interventions in male-dominated occupations are perceived of as authentic and relatable, reframe help-seeking as a strength, and support adaptive coping and emotion regulation strategies not based on suppression (64, 65).

However, the transport industry largely relies on and works within the logistics chain (ie, movement and storage of freight at all stages until reaching the consumer), which is a highly complex environment comprising multifaceted industries and stakeholders interwoven across a (time) sensitive supply chain. Failure in one link of the supply chain has a significant impact on others and can ultimately cease all operations. Therefore, a challenge unique to the transport industry is that any initiative (such as a suicide prevention gatekeeper training or workplace mental health and well-being interventions) that requires workers to come offline from the supply chain will have negative consequences for the stoppage of movement within the chain and may inadvertently contribute to workplace stress. Additionally, safety regulations for fatigue translate to specific operational and required rest hours which further impacts upon opportunities for employees to participate in programs outside of work hours. These occupational barriers have implications for how motivated organizations and workers may be for engaging in such programs, resulting in issues with feasibility and sustainability. In the maritime industry there may be more opportunity for interventions while at sea versus the highly stressful and time sensitive periods at port or during river passage, or during rest hours (70). Various recommendations have been made regarding ‘whole of ship’ training around physical and mental health and well-being, promoting resilience, and creating more supportive and equal work environments in the maritime industry (75). However, the pressures of globalized supply chain deliverables, rotating crew, and the trend toward hiring international workers at low wages and with lower standards of employment are important systemic barriers that require industry level consideration (75–78). Future research regarding suicide prevention strategies is required to determine ways to address co-occurring and interconnected contributing risk factors, and also the manner in which such initiatives can be delivered and tailored to the unique challenges of the transport sector. Industry and stakeholder involvement in the co-design of programs may be one important avenue.

### Methodological considerations

Several important considerations relate to the current findings. Across studies and samples, there were inconsistencies in how occupation/working status was defined with slight variations in conceptualization across occupational classification systems. Studies that had overly broad occupation definitions were excluded, which may mean that some transport workers were missed. Furthermore, there were differences in when occupation was assigned (ie, at the beginning of the data collection period or upon death) which may also influence the results. This, in addition to diversity across the transport samples by sector and across studies by design, may explain some of the high heterogeneity observed in this meta-analysis. There are also inherent challenges to suicide surveillance that may have impacted current findings. For example, difficulties with classifying suicide cases (79) or limited capacity for comprehensive record linkage to facilitate adequate control of known risk factors (80). Indeed, an inability to adequately control for confounders, besides age and gender, was a routinely cited limitation in the included articles. We combined general working age populations and the employed population as a reference group in the overall meta-analysis; however, subgroup analyses were performed which did not show any difference between models in which the comparator was the general or the employed population. This indicates that there was no ‘healthy worker’ effect whereby it might be expected that the general population which includes people unable to work due to illness, mental health, and physical or intellectual abilities may have an elevated risk for suicide or poor outcomes whereas the working population may be protected from such (81). A lack of a healthy worker effect was also directly acknowledged across several included articles (19, 45, 46). While our review was intended to be as comprehensive as possible on this complex and understudied topic within the diverse and highly stressful transport industry, we note that several studies were published prior to 2005. Subgroup analyses by year of publication were non-significant, however, it appears that the overall risk of suicide in transport workers may be influenced by these older studies. There have been considerable changes to society, workplace safety practices, globalization, staffing, and technology in recent years which may have had a substantial impact on the transport industry and ultimately risk for suicide. Finally, with a reliance on non-randomized and observational designs, it is difficult to assess certainty of evidence such as through GRADE assessments (82).

### Strengths and limitations

The review followed the most up to date PRISMA guidelines. Furthermore, by focusing on transport workers specifically, we were able to conduct subgroup analyses across multiple transport industries in addition to other sample and study characteristics. Nevertheless, there are certain limitations that should be noted. The review was limited to English-language publications, which may have resulted in some missing articles. Indeed, the included articles were all from high-income countries which means it is likely that our review may have missed studies from low- and middle-income countries (LMIC) and was unable to provide solid conclusions relating to suicide among transport workers from LMIC. This is important, firstly, as most suicides around the world occur in LMIC (2), and secondly, the transport industry (especially merchant seafaring) is a relatively international occupation with the potential for unique stressors in migrant or foreign workers from LMIC or those working under ‘flags of convenience’ (ie, ships registered to countries without residency requirements) (75, 77).

Another important limitation is the quality assessment tool that was used in the current study. There is a lack of consensus regarding high quality, validated quality assessment/risk of bias tools for descriptive and observational studies, so we relied upon a generic measure in the present study. While the quality assessment was conducted independently and several included studies were rated as high quality, it is acknowledged that descriptive and observational designs are more susceptible to risk of bias and findings should be interpreted in light of this (56). The sensitivity analysis removing low quality studies produced a slightly stronger effect for risk of suicide in transport workers. Subgroup analyses by study design also revealed there was a significant difference whereby cohort and case–control studies demonstrated a significant risk of suicide in transport workers whereas case series and ecological studies were not significant.

There was high heterogeneity observed in the meta-analysis which may be the result of including different reference groups, classifications of occupations and suicides, and country/regions. Finally, there were relatively few studies identified that examined suicide in the commercial aviation sector. This may reflect systemic factors described earlier (eg, fitness for work assessments) which may indicate suicides occur less frequently in these workers or it could be due to a lack of research attention. Instead, we identified, and subsequently excluded studies that involved plane-assisted suicides using light aircraft, hobby pilots, or general aviation.

### Concluding remarks

This review provides a comprehensive investigation of suicide risk among transport workers specifically. Overall, transport workers had a significantly higher risk for suicide than the general and employed populations. Based on subgroup analyses, it appears that the observed risk for suicide among transport workers relative to the general and employed populations is driven by the association for those working in maritime occupations. These findings highlight an important need for tailored suicide prevention and mental health promotion initiatives that are sensitive to organizational practices and culture in this unique, high-pressure and male-dominated industry.

## Supplementary material

Supplementary material

## Data Availability

Data can be obtained from the authors on request.
