# Supplementary material for: Suicide among transport industry workers: A systematic review and meta-analysis
Source: Scand J Work Environ Health. 2022 Oct 29;48(8):598–610. doi: 10.5271/sjweh.4059 (PMC10546617; doi:10.5271/sjweh.4059)
Supplement: Supplementary material [file SJWEH-48-598-S001.pdf]

Suicide among transport industry workers: A systematic review and meta-analysis[1]

by Sharna Mathieu, PhD,[2] Victoria Ross, PhD, Rachmania Wardhani, BPsych, Paula Brough, PhD, Darren Wishart, PhD, Xi Wen Chan, PhD, Kairi Kõlves, PhD

1. Supplementary material
2. Correspondence to: Dr Sharna Mathieu, Australian Institute for Suicide Research and Prevention, Psychology Building (M24), Griffith University Messines Ridge Road, Mount Gravatt, Queensland, 4122, Brisbane, Australia. [E-mail: s.mathieu@griffith.edu.au]

Table S1 – PRISMA Checklist

Section and Topic	Item #	Checklist item	Location where item is reported
**TITLE**			
Title	1	Identify the report as a systematic review.	Title Page
**ABSTRACT**			
Abstract	2	See the PRISMA 2020 for Abstracts checklist.	After title page
**INTRODUCTION**			
Rationale	3	Describe the rationale for the review in the context of existing knowledge.	p.1-2
Objectives	4	Provide an explicit statement of the objective(s) or question(s) the review addresses.	p.2
**METHODS**			
Eligibility criteria	5	Specify the inclusion and exclusion criteria for the review and how studies were grouped for the syntheses.	p.2
Information sources	6	Specify all databases, registers, websites, organisations, reference lists and other sources searched or consulted to identify studies. Specify the date when each source was last searched or consulted.	p.2
Search strategy	7	Present the full search strategies for all databases, registers and websites, including any filters and limits used.	p.2
Selection process	8	Specify the methods used to decide whether a study met the inclusion criteria of the review, including how many reviewers screened each record and each report retrieved, whether they worked independently, and if applicable, details of automation tools used in the process.	p.2
Data collection process	9	Specify the methods used to collect data from reports, including how many reviewers collected data from each report, whether they worked independently, any processes for obtaining or confirming data from study investigators, and if applicable, details of automation tools used in the process.	p.2-3
Data items	10a	List and define all outcomes for which data were sought. Specify whether all results that were compatible with each outcome domain in each study were sought (e.g. for all measures, time points, analyses), and if not, the methods used to decide which results to collect.	p.2-3
	10b	List and define all other variables for which data were sought (e.g. participant and intervention characteristics, funding sources). Describe any assumptions made about any missing or unclear information.	p.2-3
Study risk of bias assessment	11	Specify the methods used to assess risk of bias in the included studies, including details of the tool(s) used, how many reviewers assessed each study and whether they worked independently, and if applicable, details of automation tools used in the process.	p.2-3
Effect measures	12	Specify for each outcome the effect measure(s) (e.g. risk ratio, mean difference) used in the synthesis or presentation of results.	p.2-3
Synthesis methods	13a	Describe the processes used to decide which studies were eligible for each synthesis (e.g. tabulating the study intervention characteristics and comparing against the planned groups for each synthesis (item #5)).	p.3

Section and Topic	Item #	Checklist item	Location where item is reported
	13b	Describe any methods required to prepare the data for presentation or synthesis, such as handling of missing summary statistics, or data conversions.	p.3
	13c	Describe any methods used to tabulate or visually display results of individual studies and syntheses.	p.2-3
	13d	Describe any methods used to synthesize results and provide a rationale for the choice(s). If meta-analysis was performed, describe the model(s), method(s) to identify the presence and extent of statistical heterogeneity, and software package(s) used.	p.3
	13e	Describe any methods used to explore possible causes of heterogeneity among study results (e.g. subgroup analysis, meta-regression).	p.3
	13f	Describe any sensitivity analyses conducted to assess robustness of the synthesized results.	p.3
Reporting bias assessment	14	Describe any methods used to assess risk of bias due to missing results in a synthesis (arising from reporting biases).	NA
Certainty assessment	15	Describe any methods used to assess certainty (or confidence) in the body of evidence for an outcome.	NA (discussed p.9)
**RESULTS**			
Study selection	16a	Describe the results of the search and selection process, from the number of records identified in the search to the number of studies included in the review, ideally using a flow diagram.	p.3-4
	16b	Cite studies that might appear to meet the inclusion criteria, but which were excluded, and explain why they were excluded.	p.3-4
Study characteristics	17	Cite each included study and present its characteristics.	p.3-4
Risk of bias in studies	18	Present assessments of risk of bias for each included study.	p.4-5 (discussed p.10)
Results of individual studies	19	For all outcomes, present, for each study: (a) summary statistics for each group (where appropriate) and (b) an effect estimate and its precision (e.g. confidence/credible interval), ideally using structured tables or plots.	p.4-6
Results of syntheses	20a	For each synthesis, briefly summarise the characteristics and risk of bias among contributing studies.	Supp Table
	20b	Present results of all statistical syntheses conducted. If meta-analysis was done, present for each the summary estimate and its precision (e.g. confidence/credible interval) and measures of statistical heterogeneity. If comparing groups, describe the direction of the effect.	p.4-6
	20c	Present results of all investigations of possible causes of heterogeneity among study results.	p.4-6
	20d	Present results of all sensitivity analyses conducted to assess the robustness of the synthesized results.	p.4
Reporting biases	21	Present assessments of risk of bias due to missing results (arising from reporting biases) for each synthesis assessed.	NA
Certainty of evidence	22	Present assessments of certainty (or confidence) in the body of evidence for each outcome assessed.	NA (discussed p.9)
**DISCUSSION**			
Discussion	23a	Provide a general interpretation of the results in the context of other evidence.	p.5-10
	23b	Discuss any limitations of the evidence included in the review.	p.9
	23c	Discuss any limitations of the review processes used.	p.9-10
	23d	Discuss implications of the results for practice, policy, and future research.	p.8-9
**OTHER INFORMATION**			
Registration and protocol	24a	Provide registration information for the review, including register name and registration number, or state that the review was not registered.	p.2

Section and Topic	Item #	Checklist item	Location where item is reported
	24b	Indicate where the review protocol can be accessed, or state that a protocol was not prepared.	p.2
	24c	Describe and explain any amendments to information provided at registration or in the protocol.	NA
Support	25	Describe sources of financial or non-financial support for the review, and the role of the funders or sponsors in the review.	Before references
Competing interests	26	Declare any competing interests of review authors.	Before references
Availability of data, code and other materials	27	Report which of the following are publicly available and where they can be found: template data collection forms; data extracted from included studies; data used for all analyses; analytic code; any other materials used in the review.	Before references

From: Page MJ, McKenzie JE, Bossuyt PM, Boutron I, Hoffmann TC, Mulrow CD, et al. The PRISMA 2020 statement: an updated guideline for reporting systematic reviews. BMJ 2021;372:n71. doi: 10.1136/bmj.n71

Table S2 Overview and characteristics of studies included in the review (*n*=23)

Authors & Year	Transport Sector	Occupations	Country	Data Period & Sample	Occupational Classification/ Definition	Suicide definition	Design	Effect Measure and/or analysis	Sample size(s)	Comparison/ Population or Reference Group	Quality Rating♦
**Agerbo et al 2007**	General transport/ mixed	Drivers and mobile plant operators; Motor vehicle drivers; Transport and other labourers	Denmark	1991-1997; Danish residents aged 15-60 (i.e., working age) who died by suicide. Must have resided in Denmark throughout the two preceding years.	Danish version of the International Classification of Occupations (DISCO-88).	ICD-8 (E950-E959); ICD-10 (X60-X84). NOT including 'open verdicts'.	Nested case-control study	Rate Ratios (RR); analysed using conditional logistic regression.	N=3195 (suicide cases, n=898 females) N=63900 controls	A random sample of 20 controls for each person who died by suicide, of the same gender who were born in the same year and who were alive when the case died by suicide.<br><br>Primary education teaching professionals chosen as 'reference category' in analysis because this occupation is relatively large, well-defined, and universal.	19 (High)
**Andersen et al (2010)**	General transport/ mixed	Transport workers (including drivers, road and rail drivers)	Australia (QLD)	1990-2006; All suicides across occupational groups in QLD (15-64 years)	Australian Standard Classification of Occupations (ASCO-2)	Based on Decision Tree model, identified as 'probable' and 'beyond reasonable doubt'	Case series	Crude suicide rate (calculated using ABS data for 2001); Rate ratios with 95%CI	7652 (total cases identified as 'probable' and 'beyond reasonable doubt' aged 15-64 years; n=6087 males)<br><br>n=3010 ('Employed Population')	Employed population (aged 15-64 years) in QLD	18 (High)

									were currently employed at time of death (n=1960 had occupation other than those of interest, and 123 could not be identified).		
**Ballard (2002)**	Aviation	Cockpit crew, cabin attendants	Italy	1 January 1965-31 December 1996 (observation period); and follow-up procedure between January 1999 and June 2000; all Italian-born flight personnel in active service with the Italian national airline.	Airline personnel records: Cockpit crews and cabin attendants.	ICD-8 and ICD-9 classification of underlying causes of death (depending on time of death and revision of ICD system in use)	Retrospective cohort study	SMR with exact 95% CI calculated from exact Poisson probabilities	Male cockpit crew members (n=3022), male flight cabin flight attendants (n=3418), female cabin attendants (n=3428)	Italian general population	15 (Med)
**Brandt et al (1994)**	Maritime	Merchant seafarers (Deck crew and officers, Engine crew and officers)	Denmark	1970-1985; Danish male seafarers	Seamen identified from census, including those at sick-leave or occasionally unemployed; and seafarers as registered by the Danish Seafarers Tax Office.	ICD-8 (E950-E959)	Retrospective cohort study	Mortality Rate Ratios (MRR) and adjusted MRRs.	1,292,337 all men enrolled. 116,368 total deaths. Deck officers: 3906 (50542 person years) and 395 deaths Deck crew: 5646 (75228 person years) and 594 deaths. Engine officers: 3442 (45426 person years), 340 deaths Engine crew: 977 (11901 person years), 174 deaths.	Men aged 20-65 years who were 'economically active' in 1970	18 (High)
**Guidotti (1992)**	Transit/ Driving	Bus drivers	Canada (Alberta)	1927-1985/1987; bus drivers of Edmonton Transit who either died while employed	Based on Transit Union (ATU) Local 569, which	ICD-9 (950-959); and 'suicide by	Retrospective cohort study	Proportionate Mortality Ratio (PMR)	219 (total deaths all males). However,	General male population of the Alberta province	13 (Med)

				or retired after 20 years of service who are registered for the union's death benefits programme.	represents around 1400 workers, mostly bus drivers in the city of Edmonton.	gas poisoning' (ICD-9 951-952)			death certificates obtained for 216, between 1927-1987 Median age at death: 71 years (range: 20-99 years).	('non-Indians', as 'there have been few non-white transit workers in this union')	
**Guseva Canu et al 2019**	General transport/ mixed	Ship and aircraft controllers/ technicians, Material recording and transport clerks, Locomotive engine drivers and related, motor vehicle drivers, garbage collectors and related	Switzerland	1990 & 2000-2014; adults aged 18-65 years registered in the 1990 or 2000 census and the Swiss National Cohort	Nomenclature Suisse des Professions (NSP/Swiss classification of occupations) RECODED into ISCO -88	For deaths from 1990-1994 = ICD-8 (950-958); For deaths from 1995 onwards = ICD-10 (X60-X84)	Prospective cohort study	Direct age-standardized mortality rates (DSR) and SMR	N = 5834618 (49% women)<br>At study endpoint, n = 238504 had died, n = 19863 of whom by suicide	General Swiss adult population (General population of working age)	19 (High)
**Hansen & Jensen 1998**	Maritime	Seafaring/ navigation and radio/ ship engineer	Denmark	1986-1993; Danish women employed as a seafarer during the study period	Danish Maritime Authority Personnel Records	ICD-8 (E950-959)	Retrospective cohort study	Standardised mortality ratio (SMR)	N = 6788 total cohort; n = 9 suicides of which n = 2 were in a traditional seafaring role (n=7 in food preparation not extracted for this study)	General population of Danish women (age and specific mortality)	14 (Med)
**Kposowa et al 1999**	General transport/ mixed	Transport equipment operatives	USA	1979-1989; individuals aged 15 and above, non-Hispanic whites, non-Hispanic African Americans, and Hispanic males and females. (noninstitutionalised)	Unclear classification system.<br>(A list of industries and sub-occupations were derived and coded)	ICD-9 (E950-E959)	Prospective cohort study	Hazard Ratio (but defined as relative risk/ RR) (using Cox model)	N = 471922, n suicides = 545	Farmers and farm managers	18 (High)

**Labovitz & Hagedorn, 1971****	General transport/ mixed	Locomotive engineers, Motormen Street subway and elevated railway, taxicab and chauffeurs, truck and tractor drivers, delivery and routemen	USA	1950; US males employed in 36 occupations (occupations that have sufficient data on suicide)	Duncan's comparisons of occupational categories used in 1947 survey to the detailed occupational classification in the US Census 1950.	Unclear	Case series	Suicide rates, Spearman's rho and Pearson's *r*	NR	Unclear	8 (Low)
**Meltzer et al 2008***	Transit/ Driving	Van drivers	England & Wales	2001-2005; men and women aged 20-64 years	Standard Occupational Classification 2000 (SOC 2000)	ICD-10	Case series	Proportional Mortality Ratio (PMR) and Standardised Mortality Ratio (SMR)	n suicides in all occupation groups: 14332	General population of England and Wales	14 (Med)
**Milner et al (2015)***	Transit/ Driving	Road and rail drivers	Australia	2001-2010; All deaths recorded as intentional self-harm in males in driving occupations (road and rail)	Australian and New Zealand Standard Classification of Occupations sub-group major group '73-Road and Rail drivers'	Cases officially recorded as 'intentional self-harm' in NCIS.	Case series	Age standardised suicide rates per 100,000; Incidence rate ratios (IRR) with 95% CI using Mantell Haenszel rates	26471 total individual cases; 513 suicides among Road and Rail drivers (n=502/ 98% males -- only males included in analysis).	General working population in all other occupations coded according to ANZSCO.	18 (High)
**Milner et al (2017)***	Transit/ Driving	Access to carbon monoxide (Automobile drivers, bus and coach drivers, delivery drivers, truck drivers, vehicle body builders/ trimmers and painters, panel beaters and automotive engineering	Australia	2001-2012; all employed adults with a known occupation who were 15 years of age or older at the time of death.	Australian and New Zealand Standard Classification of Occupations (ANZSCO) - four-digit level.	ICD-10 (X60-X84)	Case series	Rates per 100,000 persons; negative binomial regression (coefficients in Incidence Rate Ratios/ IRRs)	N = 10150 (n=8600 males; n=1550 females)	Population estimates of different ANZSCO occupational groups based on 2006 census data (mid-point of study)	18 (High)

**Mustard et al 2010**	General transport/ mixed	Transport operators (air, rail, water, motor, other)	Canada	1991-2001; occupationally active and inactive men and women aged 30-69 years at baseline which represents 15% of population	1980 Standard Occupation Classifications (for occupation groups --80 minor groups)	ICD-9 for deaths occurring from 1991-1999; ICD-10 for deaths occurring from 2000-2001	Prospective cohort study	Crude mortality rate, adjusted-SMR (direct method), and standardised Risk Ratio (SRR)	occupationally active men n = 929300<br>occupationally active women n = 774800<br>occupationally active N = 1704100<br>occupationally inactive men n = 133100<br>occupationally inactive women n = 264300<br>occupationally inactive N = 397500<br>n=1932 suicides in occupationally active men; n=424 suicides in non-active men & n=428 suicides in occupationally active women, n=227 in non-active women	All occupationally active and inactive men and women (Mid-year population estimates from 1991)	17 (High)
**Rafnasson & Gunnarsdottir (1993)**	Maritime	Merchant seamen (including fishermen and merchant sailors)	Iceland	1966-1989; seamen (including fishermen and merchant sailors)	Based on membership of a seamen pension fund from 1958 to 1986.	ICD-7 (E963, E970-E979)	Retrospective cohort study	Standardised mortality ratios (SMRs) with 95% Cis assuming a Poisson distribution; Spearman's rank correlation	27884 seamen: 2226 (total deaths): 144 (suicide deaths)	General population	14 (Med)

								with one-tailed p-values.			
**Roberts et al (2013)**	General transport/ mixed	Forklift drivers, dockers and stevedores, rail transport operators, driving instructors, merchant seafarers	England & Wales	1979-1980, 1982-1983, and 2001-2005; suicide deaths across all occupations	The 2000 Standard Occupational Classification (SOC 2000) (for 2001-2005 period); the 1980 International Standard Classification of Occupation (ISCO-1980) (for 1979-1980 and 1982-1983 period).	ICD-9 (E980-E989, E950-E959) for 1979-1980 and 1982-1983 period; ICD-10 (Y10-33.8, Y34, X60-X84) for 2001-2005.	Case series	Suicide rate per 100,000; Standardised Mortality Ratios (SMRs), Proportional Mortality Ratios (PMRs)	Unclear - the study did not report total N, and only presented results for 50 'high risk occupations' and 30 'highest risk occupations'.<br><br>Occupations with small number of suicides (<5) were excluded and the actual numbers were not reported.	General British population (age-matched)	11 (Low)
**Schmid et al 2020***	General transport/ mixed	Drivers and mobile plant operators, transport and other labourers	Switzerland	1990-2014; all adults aged 18-65 at the 1990 or 2000 census, excluding unemployed, job-seeking, and those with unknown occupation (sample 1); those deceased by suicide within each occupation (sample 2)	Swiss classification of occupations recoded into four-digit ISCO 88; for this study the two-digit ISCO 88 was used.	Deaths from 1990-1994 = ICD-8 (950-958).<br><br>Deaths from 1995 onwards = ICD-10 (X60-X84).<br><br>Only including cases where suicides were 'principal cause' of death, EXCLUDING assisted suicide	Prospective cohort study	Standardised mortality ratios (SMR)	N = 5834618 (49% women)<br><br>n= 19863 died by suicide (sample 1)<br><br>n suicides with available occupation = 13331 (sample 2)	Swiss general population	18 (High)
**Stack, 2001****	Transit/ Driving	Truck drivers	USA (21 states)	1990; working men and women in 32 selected occupations	Standard federal occupational codes (US Public Health Service, 1994); selected occupations with having at least 50	Coroner's report, unclear 'definition'.	Case series	Odds Ratio; bivariate and multivariate logistic regression	n suicides = 9499, n deaths from other causes = 127687	The rest of working-age population (Deaths by all other causes during the study period in working age population.)	9 (Low)

					suicides (some occupations were combined to have enough suicides)						
**Stallones et al 2013**	General transport/ mixed	Transportation and material moving	USA (Colorado)	2004-2006; white men and women in Colorado aged 16 and above	2000 Standard Occupation Classifications	ICD-10	Case series	Age-adjusted suicide rates per 100,000	N=2352 suicides<br>n Males = 1536<br>n Females = 337	US 2000 population (as standard population)	16 (Med)
**Too et al (2020)**	Transit/ Driving	Road and rail drivers	Australia	2001-2016; all individuals died by suicide who were employed at time of death.	Australian and New Zealand Standard Classification of Occupations)	ICD-10 (X60-X84)	Ecological	Suicide rates (using 2011 Census data); spatial, temporal, and spatial-temporal suicide clusters analysis; Poisson discrete scan statistic; Monte Carlo stimulation	14317 (total deaths by suicide across all occupation groups)	Population estimates by occupation by postal code obtained from the Australian Bureau of Statistics 2011	17 (High)
**Wada et al 2016**	General transport/ mixed	Transport and machine operation	Japan	2010; Japanese men of working age (25-59 years old)	Based on reports from the deceased person's family, recorded in the vital statistics.	ICD-10	Case series	Age-adjusted incident relative risk (IRR)	Suicide deaths N = 11551.<br>Excluded n (not employed) = 4356; n(without occupational data) = 2297; n (without industry data) = 2507.<br>N analysed = 4898 (occupations), and 4688 (industries)	Sales occupation	16 (Med)

**Windsor-Shellard & Gunnell 2019**	General transport/ mixed	Forklift truck drivers	England	2011-2015; individuals aged 20-64 years for each occupation category (employed at time of death; or out of work due to retirement, being unemployed, or being sick--based on last occupation)	Standard Occupation Classification (SOC2010) - 9 major categories and 350+ individual occupations included	ICD-10 (X60-X84)	Case series	Indirect-Standardised Mortality Ratio (Indirect SMR); restricted analysis to occupations with at least 20 deaths	13232 suicide deaths among people aged 20-64 years who had an occupation recorded on the death certificate; 81% (n=10688) men.	General population/ all 'usual residents of England' aged 20-64 years.	19 (High)
**Wild et al 2021***	General transport/ mixed	Motor vehicle drivers, transport and other labourers	Switzerland	1990-2014; working women aged 18-65 living in Switzerland	4-digit ISCO 1988 (originally), but then aggregated using the 2 digits.	Not described but uses a cohort described in Guseva Canu et al 2019 & Schmid et al 2020	Prospective cohort study	Adjusted RRs	Total women N = 1771940<br><br>n = 2526 deaths by suicide (overall suicide rate: 10.1/100,000)	One occupation = office clerks (for calculating RR); One sector = Trade, repair car/domestic articles; All other workers	18 (High)
**Yamauchi et al (2018)**	General transport/ mixed	Transport and postal	Japan	January 2010-March 2015; cases involving compensation for mental disorders and suicides across occupation groups	Japan Standard Industrial Classification (JSIC) by the Ministry of Internal Affairs and Communication	Based on 'medicolegal examinations by licensed physicians and police investigations' (for compensation claims purpose)	Case series	Incidence Rates (IR) per 1 million employees.	1990 (total included subjects) --> 379 suicide cases.<br>17 women (4%), 362 men (96%)	n/a	12 (Low)

NB: n/a = not analysed or not applicable; nr = not reported; IR = incidence rates; SMR = standardised mortality ratio; CMR = crude mortality ratio; PMR = proportional mortality ratio; ICD = international classification of diseases; 95% CI = 95% confidence interval; RR = rate ratio; IRR = incidence rate ratio; MRR = mortality rate ratio

* Not included in any of the final meta-analyses given overlap in data with more suitable articles

** Not included in any of the final meta-analyses given insufficient information for calculating effects compared to the general or employed population

♦ Quality assessment is based upon a modified version (Reardon et al., 2017) of a generic quality appraisal tool suitable for the array of study designs identified in this study developed by Kmet and colleagues (2004).

Table S3 Overview of the final quality assessment (*n*=23)

**Study**	**Question/ Objective sufficiently described?**	**Study design evident and appropriate?**	**Method of subject/ comparison group selection or source of information/ input variables described and appropriate?**	**Subject (and comparison group, if applicable) characteristics sufficiently described?**	**Outcome and exposure measure(s) well defined and robust to measurement / misclassification bias? Means of assessment reported?**	**Sample size appropriate?**	**Analytic methods described/ justified and appropriate?**	**Controlled confounders?**	**Results reported in sufficient detail?**	**Conclusions supported by the results?**	**Total**
**Agerbo et al 2007**	2	2	2	2	2	2	2	2	1	2	19
**Andersen et al 2010**	2	2	2	2	1	2	2	1	2	2	18
**Ballard et al 2002**	1	2	1	1	2	1	2	1	2	2	15
**Brandt et al 1994**	1	2	2	2	2	2	2	1	2	2	18
**Guidotti 1992**	2	1	1	1	1	1	1	1	2	2	13
**Guseva Canu et al. 2019**	2	2	2	2	2	2	2	1	2	2	19
**Hansen & Jensen 1998**	2	2	2	0	2	1	2	1	1	1	14
**Kposowa, 1999**	2	2	2	1	2	2	2	1	2	2	18
**Labovitz et al 1971****	1	1	0	1	1	0	1	0	1	2	8
**Meltzer et al. 2008***	2	2	1	1	2	1	2	1	1	1	14
**Milner et al 2015***	2	2	2	2	1	2	2	1	2	2	18
**Milner et al 2017***	2	2	1	2	2	2	1	2	2	2	18
**Mustard et al. 2010**	2	2	2	2	2	2	1	1	1	2	17
**Rafnasson et al 1993**	2	2	1	1	2	1	1	1	1	2	14
**Roberts et al 2013**	2	1	1	0	2	0	1	1	1	2	11
**Too et al 2020**	2	2	2	1	2	2	2	1	1	2	17
**Schmid et al. 2020***	2	2	2	2	2	2	2	0	2	2	18
**Stack, 2001****	1	1	1	0	0	1	1	1	1	2	9
**Stallones et al. 2013**	2	2	1	2	2	1	1	1	2	2	16
**Wada et al. 2016**	2	2	1	1	2	2	2	1	1	2	16
**Wild et al. 2021***	2	2	2	2	1	2	2	1	2	2	18

**Windsor-Shellard et al. 2019**	2	2	1	2	2	2	2	2	2	2	19
**Yamauchi et al 2018**	2	2	1	2	1	1	1	0	1	1	12

NB: Quality assessment is based upon a modified version (Reardon et al., 2017) of a generic quality appraisal tool suitable for the array of study designs identified in this study developed by Kmet and colleagues (2004).

* Not included in any of the final meta-analyses given overlap in data with more suitable articles

** Not included in any of the final meta-analyses given insufficient information for calculating effects compared to the general or employed population

Supplementary Figures – Subgroup Analyses

A. By Gender

Study or Subgroup	log[Effect Size]	SE	Weight	Effect Size IV, Random, 95% CI
**1.2.1 Males**				
Andersen 2010 (general transport, males)	0.207	0.0856	2.5%	1.23 [1.04, 1.45]
Ballard 2002 (cabin attendant, males)	-0.5108	0.8212	0.5%	0.60 [0.12, 3.00]
Ballard 2002 (cockpit crew, males)	0.4574	0.3734	1.5%	1.58 [0.76, 3.28]
Brandt 1994 (deck crew, 20-34y, males)	0.9821	0.2333	2.0%	2.67 [1.69, 4.22]
Brandt 1994 (deck crew, 35-49y, males)	0.4318	0.2306	2.0%	1.54 [0.98, 2.42]
Brandt 1994 (deck crew, 50-64y, males)	0.4886	0.3322	1.6%	1.63 [0.85, 3.13]
Brandt 1994 (deck officers, 20-34y, males)	0.0198	0.2221	2.0%	1.02 [0.66, 1.58]
Brandt 1994 (deck officers, 35-49y, males)	0.3577	0.2776	1.8%	1.43 [0.83, 2.46]
Brandt 1994 (deck officers, 50-64y, males)	-0.9676	0.6811	0.7%	0.38 [0.10, 1.44]
Brandt 1994 (engine crew, 20-34y, males)	2.0281	0.5818	0.9%	7.60 [2.43, 23.77]
Brandt 1994 (engine crew, 35-49y, males)	1.4816	0.2684	1.9%	4.40 [2.60, 7.45]
Brandt 1994 (engine crew, 50-64y, males)	1.1787	0.1854	2.2%	3.25 [2.26, 4.67]
Brandt 1994 (engine officers, 20-34y, males)	0.8755	0.4782	1.1%	2.40 [0.94, 6.13]
Brandt 1994 (engine officers, 35-49y, males)	-0.3285	0.4137	1.3%	0.72 [0.32, 1.62]
Brandt 1994 (engine officers, 50-64y, males)	-0.462	0.6993	0.7%	0.63 [0.16, 2.48]
Guidotti 1992 (bus drivers, males)	0.2647	0.358	1.5%	1.30 [0.65, 2.63]
Guseva Canu 2019 (garbage collectors, males)	0.4187	0.2731	1.8%	1.52 [0.89, 2.60]
Guseva Canu 2019 (locomotive eng drivers, males)	-0.1863	0.1326	2.4%	0.83 [0.64, 1.08]
Guseva Canu 2019 (motor vehicle drivers, males)	0.1484	0.0508	2.6%	1.16 [1.05, 1.28]
Guseva Canu 2019 (ship, air control&tech, males)	-0.0943	0.2477	1.9%	0.91 [0.56, 1.48]
Guseva Canu2019(transport clerks,recording, males)	0.1398	0.0562	2.6%	1.15 [1.03, 1.28]
Mustard 2010 (motor transport, males)	0.3192	0.0938	2.5%	1.38 [1.14, 1.65]
Mustard 2010 (railway transport, males)	-0.3696	0.4139	1.3%	0.69 [0.31, 1.56]
Rafnsson 1993 (seamen, males)	0.3293	0.0836	2.5%	1.39 [1.18, 1.64]
Stallones 2013 (transport, material moving, males)	-0.0655	0.0257	2.6%	0.94 [0.89, 0.98]
Wada 2016 (transport, machine operation, males)	0.4574	0.1236	2.4%	1.58 [1.24, 2.01]
Windsor-Shellard 2019 (forklift drivers, males)	0.4511	0.1163	2.4%	1.57 [1.25, 1.97]
Yamauchi 2018 (transport&postal, 30-39y, males)	0.2683	0.3272	1.6%	1.31 [0.69, 2.48]
Yamauchi 2018 (transport&postal, 40-49y, males)	-0.0339	0.3258	1.6%	0.97 [0.51, 1.83]
Yamauchi 2018 (transport&postal, 50-59y, males)	-0.499	0.4596	1.2%	0.61 [0.25, 1.49]
Yamauchi 2018 (transport&postal, over 60y, males)	-0.2231	0.7506	0.6%	0.80 [0.18, 3.48]
Yamauchi 2018 (transport&postal, under30y, males)	-0.1744	0.5927	0.9%	0.84 [0.26, 2.68]
**Subtotal (95% CI)**			**55.4%**	**1.34 [1.18, 1.52]**
Heterogeneity: Tau² = 0.07; Chi² = 183.50, df = 31 (P < 0.00001); I² = 83%				
Test for overall effect: Z = 4.42 (P < 0.0001)				
**1.2.2 Females**				
Ballard 2002 (cabin attendant, females)	1.2179	0.5116	1.1%	3.38 [1.24, 9.21]
Guseva Canu 2019 (motor vehicle drivers, females)	0.6831	0.3384	1.6%	1.98 [1.02, 3.84]
Guseva Canu2019(transportclerks,recording,females)	0.0953	0.238	2.0%	1.10 [0.69, 1.75]
Hansen 1998 (seafarers, females)	1.7334	1.0737	0.4%	5.66 [0.69, 46.42]
Stallones 2013 (transport,material moving,females)	-0.5225	0.0571	2.6%	0.59 [0.53, 0.66]
**Subtotal (95% CI)**			**7.5%**	**1.48 [0.72, 3.05]**
Heterogeneity: Tau² = 0.49; Chi² = 32.75, df = 4 (P < 0.00001); I² = 88%				
Test for overall effect: Z = 1.07 (P = 0.28)				
**1.2.3 All Persons**				
Agerbo 2007 (drivers&mobile plant operators, all)	0.5596	0.2187	2.1%	1.75 [1.14, 2.69]
Agerbo 2007 (motor-vehicle drivers, all genders)	0.3001	0.1793	2.2%	1.35 [0.95, 1.92]
Agerbo 2007 (transport and other labourers, all)	0.1655	0.2312	2.0%	1.18 [0.75, 1.86]
Andersen 2010 (general transport, all genders)	0.5878	0.0863	2.5%	1.80 [1.52, 2.13]
Kposowa 1999 (transport equip operatives, all)	0.5878	0.4267	1.3%	1.80 [0.78, 4.15]
Roberts 2013a(dockers and stevedores, all genders)	-0.1905	0.2092	2.1%	0.83 [0.55, 1.25]
Roberts 2013a (driving instructors, all genders)	-0.0827	0.2505	1.9%	0.92 [0.56, 1.50]
Roberts 2013a(forklift truck drivers, all genders)	-0.4112	0.1371	2.4%	0.66 [0.51, 0.87]
Roberts 2013a (merchant seafarers, all genders)	1.1398	0.0886	2.5%	3.13 [2.63, 3.72]
Roberts 2013a (rail operating staff, all genders)	-0.2893	0.1251	2.4%	0.75 [0.59, 0.96]
Roberts 2013b(dockers and stevedores, all genders)	0.3498	0.2587	1.9%	1.42 [0.85, 2.36]
Roberts 2013b (driving instructors, all genders)	-0.6938	0.2677	1.9%	0.50 [0.30, 0.84]
Roberts 2013b(forklift truck drivers, all genders)	0.2667	0.0954	2.5%	1.31 [1.08, 1.57]
Roberts 2013b (merchant seafarers, all genders)	1.1692	0.1722	2.2%	3.22 [2.30, 4.51]
Roberts 2013b (rail operating staff, all genders)	0.0851	0.23	2.0%	1.09 [0.69, 1.71]
Too 2020 (mobile plant operators, all genders)	0.1875	0.0734	2.5%	1.21 [1.04, 1.39]
Too 2020 (road and rail drivers, all genders)	0.7303	0.0361	2.6%	2.08 [1.93, 2.23]
**Subtotal (95% CI)**			**37.0%**	**1.32 [1.04, 1.66]**
Heterogeneity: Tau² = 0.20; Chi² = 258.93, df = 16 (P < 0.00001); I² = 94%				
Test for overall effect: Z = 2.33 (P = 0.02)				
**Total (95% CI)**			**100.0%**	**1.32 [1.16, 1.51]**
Heterogeneity: Tau² = 0.18; Chi² = 825.70, df = 53 (P < 0.00001); I² = 94%				
Test for overall effect: Z = 4.09 (P < 0.0001)				
Test for subgroup differences: Chi² = 0.10, df = 2 (P = 0.95), I² = 0%				

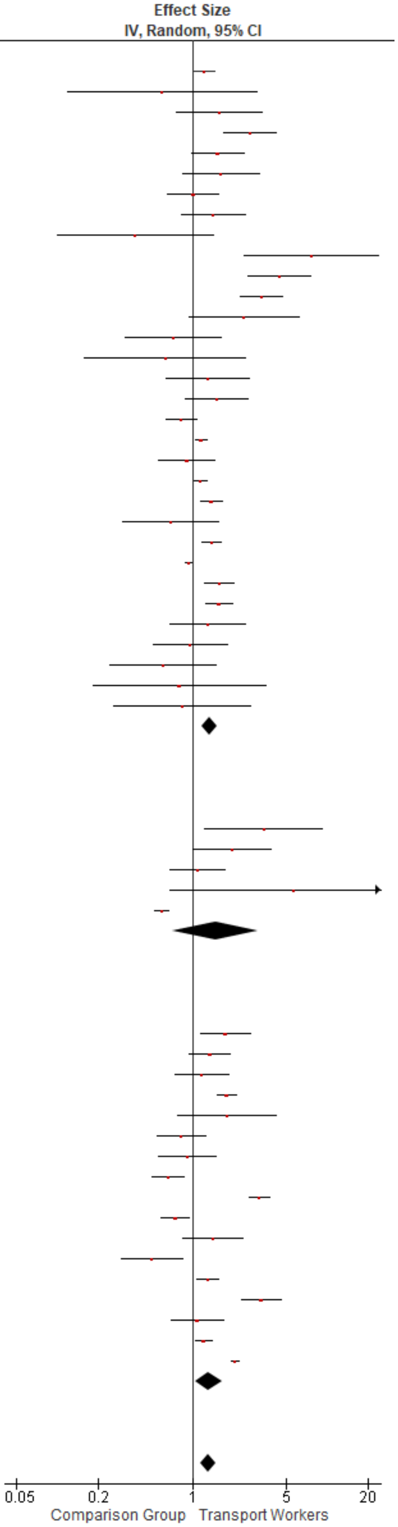

B. By Transport sector

Study or Subgroup	log[Effect Size]	SE	Weight	Effect Size IV, Random, 95% CI
**1.3.1 General or Mixed Transport Sectors**				
Agerbo 2007 (transport and other labourers, all)	0.1655	0.2312	2.1%	1.18 [0.75, 1.86]
Andersen 2010 (general transport, all genders)	0.5878	0.0863	2.5%	1.80 [1.52, 2.13]
Guseva Canu 2019 (garbage collectors, males)	0.4187	0.2731	1.9%	1.52 [0.89, 2.60]
Guseva Canu 2019 (ship, air control&tech, males)	-0.0943	0.2477	2.0%	0.91 [0.56, 1.48]
Guseva Canu2019(transportclerks,recording,females)	0.0953	0.238	2.0%	1.10 [0.69, 1.75]
Guseva Canu2019(transport clerks,recording, males)	0.1398	0.0562	2.6%	1.15 [1.03, 1.28]
Kposowa 1999 (transport equip operatives, all)	0.5878	0.4267	1.4%	1.80 [0.78, 4.15]
Stallones 2013 (transport,material moving,females)	-0.5225	0.0571	2.6%	0.59 [0.53, 0.66]
Stallones 2013 (transport, material moving, males)	-0.1507	0.0257	2.6%	0.86 [0.82, 0.90]
Too 2020 (mobile plant operators, all genders)	0.1875	0.0734	2.5%	1.21 [1.04, 1.39]
Wada 2016 (transport, machine operation, males)	0.4574	0.1236	2.4%	1.58 [1.24, 2.01]
Yamauchi 2018 (transport&postal, 30-39y, males)	0.2683	0.3272	1.7%	1.31 [0.69, 2.48]
Yamauchi 2018 (transport&postal, 40-49y, males)	-0.0339	0.3258	1.7%	0.97 [0.51, 1.83]
Yamauchi 2018 (transport&postal, 50-59y, males)	-0.499	0.4596	1.3%	0.61 [0.25, 1.49]
Yamauchi 2018 (transport&postal, over 60y, males)	-0.2231	0.7506	0.7%	0.80 [0.18, 3.48]
Yamauchi 2018 (transport&postal, under30y, males)	-0.1744	0.5927	1.0%	0.84 [0.26, 2.68]
**Subtotal (95% CI)**			**30.9%**	**1.11 [0.91, 1.35]**
Heterogeneity: Tau² = 0.10; Chi² = 184.33, df = 15 (P < 0.00001); I² = 92%				
Test for overall effect: Z = 1.02 (P = 0.31)				
**1.3.2 Transit and Driving**				
Agerbo 2007 (drivers&mobile plant operators, all)	0.5596	0.2187	2.1%	1.75 [1.14, 2.69]
Agerbo 2007 (motor-vehicle drivers, all genders)	0.3001	0.1793	2.3%	1.35 [0.95, 1.92]
Guidotti 1992 (bus drivers, males)	0.2647	0.358	1.6%	1.30 [0.65, 2.63]
Guseva Canu 2019 (locomotive eng drivers, males)	-0.1863	0.1326	2.4%	0.83 [0.64, 1.08]
Guseva Canu 2019 (motor vehicle drivers, females)	0.6831	0.3384	1.7%	1.98 [1.02, 3.84]
Guseva Canu 2019 (motor vehicle drivers, males)	0.1484	0.0508	2.6%	1.16 [1.05, 1.28]
Mustard 2010 (motor transport, males)	0.3192	0.0938	2.5%	1.38 [1.14, 1.65]
Mustard 2010 (railway transport, males)	-0.3696	0.4139	1.4%	0.69 [0.31, 1.56]
Roberts 2013a (driving instructors, all genders)	-0.0827	0.2505	2.0%	0.92 [0.56, 1.50]
Roberts 2013a(forklift truck drivers, all genders)	-0.4112	0.1371	2.4%	0.66 [0.51, 0.87]
Roberts 2013a (rail operating staff, all genders)	-0.2893	0.1251	2.4%	0.75 [0.59, 0.96]
Roberts 2013b (driving instructors, all genders)	-0.6938	0.2677	1.9%	0.50 [0.30, 0.84]
Roberts 2013b(forklift truck drivers, all genders)	0.2667	0.0954	2.5%	1.31 [1.08, 1.57]
Roberts 2013b (rail operating staff, all genders)	0.0851	0.23	2.1%	1.09 [0.69, 1.71]
Too 2020 (road and rail drivers, all genders)	0.7303	0.0361	2.6%	2.08 [1.93, 2.23]
Windsor-Shellard 2019 (forklift drivers, males)	0.4511	0.1163	2.4%	1.57 [1.25, 1.97]
**Subtotal (95% CI)**			**34.8%**	**1.13 [0.91, 1.41]**
Heterogeneity: Tau² = 0.16; Chi² = 223.32, df = 15 (P < 0.00001); I² = 93%				
Test for overall effect: Z = 1.12 (P = 0.26)				
**1.3.3 Maritime**				
Brandt 1994 (deck crew, 20-34y, males)	0.9821	0.2333	2.1%	2.67 [1.69, 4.22]
Brandt 1994 (deck crew, 35-49y, males)	0.4318	0.2306	2.1%	1.54 [0.98, 2.42]
Brandt 1994 (deck crew, 50-64y, males)	0.4886	0.3322	1.7%	1.63 [0.85, 3.13]
Brandt 1994 (deck officers, 20-34y, males)	0.0198	0.2221	2.1%	1.02 [0.66, 1.58]
Brandt 1994 (deck officers, 35-49y, males)	0.3577	0.2776	1.9%	1.43 [0.83, 2.46]
Brandt 1994 (deck officers, 50-64y, males)	-0.9676	0.6811	0.8%	0.38 [0.10, 1.44]
Brandt 1994 (engine crew, 20-34y, males)	2.0281	0.5818	1.0%	7.60 [2.43, 23.77]
Brandt 1994 (engine crew, 35-49y, males)	1.4816	0.2684	1.9%	4.40 [2.60, 7.45]
Brandt 1994 (engine crew, 50-64y, males)	1.1787	0.1854	2.2%	3.25 [2.26, 4.67]
Brandt 1994 (engine officers, 20-34y, males)	0.8755	0.4782	1.2%	2.40 [0.94, 6.13]
Brandt 1994 (engine officers, 35-49y, males)	-0.3285	0.4137	1.4%	0.72 [0.32, 1.62]
Brandt 1994 (engine officers, 50-64y, males)	-0.462	0.6993	0.8%	0.63 [0.16, 2.48]
Hansen 1998 (seafarers, females)	1.7334	1.0737	0.4%	5.66 [0.69, 46.42]
Rafnsson 1993 (seamen, males)	0.3293	0.0836	2.5%	1.39 [1.18, 1.64]
Roberts 2013a(dockers and stevedores, all genders)	-0.1905	0.2092	2.1%	0.83 [0.55, 1.25]
Roberts 2013a (merchant seafarers, all genders)	1.1398	0.0886	2.5%	3.13 [2.63, 3.72]
Roberts 2013b(dockers and stevedores, all genders)	0.3498	0.2587	2.0%	1.42 [0.85, 2.36]
Roberts 2013b (merchant seafarers, all genders)	1.1692	0.1722	2.3%	3.22 [2.30, 4.51]
**Subtotal (95% CI)**			**30.9%**	**1.82 [1.37, 2.42]**
Heterogeneity: Tau² = 0.26; Chi² = 122.01, df = 17 (P < 0.00001); I² = 86%				
Test for overall effect: Z = 4.15 (P < 0.0001)				
**1.3.4 Aviation**				
Ballard 2002 (cabin attendant, females)	1.2179	0.5116	1.1%	3.38 [1.24, 9.21]
Ballard 2002 (cabin attendant, males)	-0.5108	0.8212	0.6%	0.60 [0.12, 3.00]
Ballard 2002 (cockpit crew, males)	0.4574	0.3734	1.5%	1.58 [0.76, 3.28]
**Subtotal (95% CI)**			**3.3%**	**1.72 [0.78, 3.78]**
Heterogeneity: Tau² = 0.20; Chi² = 3.43, df = 2 (P = 0.18); I² = 42%				
Test for overall effect: Z = 1.35 (P = 0.18)				
**Total (95% CI)**			**100.0%**	**1.32 [1.15, 1.52]**
Heterogeneity: Tau² = 0.20; Chi² = 901.02, df = 52 (P < 0.00001); I² = 94%				
Test for overall effect: Z = 3.83 (P = 0.0001)				
Test for subgroup differences: Chi² = 9.66, df = 3 (P = 0.02), I² = 69.0%				

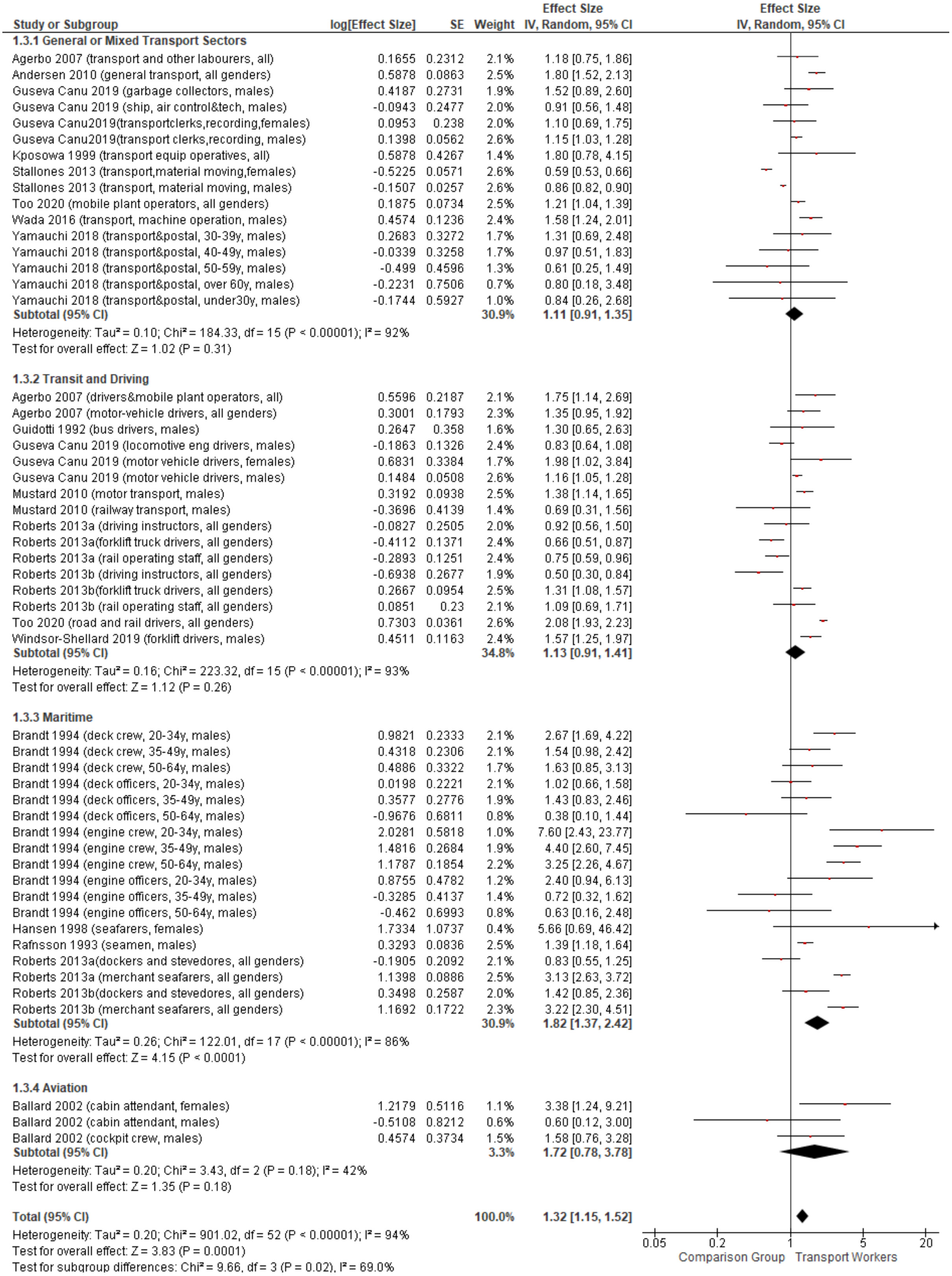

C. By Geographical Region

Study or Subgroup	log[Effect Size]	SE	Weight	Effect Size IV, Random, 95% CI
**1.4.1 Europe**				
Agerbo 2007 (drivers&mobile plant operators, all)	0.5596	0.2187	2.1%	1.75 [1.14, 2.69]
Agerbo 2007 (motor-vehicle drivers, all genders)	0.3001	0.1793	2.3%	1.35 [0.95, 1.92]
Agerbo 2007 (transport and other labourers, all)	0.1655	0.2312	2.1%	1.18 [0.75, 1.86]
Ballard 2002 (cabin attendant, females)	1.2179	0.5116	1.1%	3.38 [1.24, 9.21]
Ballard 2002 (cabin attendant, males)	-0.5108	0.8212	0.6%	0.60 [0.12, 3.00]
Ballard 2002 (cockpit crew, males)	0.4574	0.3734	1.5%	1.58 [0.76, 3.28]
Brandt 1994 (deck crew, 20-34y, males)	0.9821	0.2333	2.1%	2.67 [1.69, 4.22]
Brandt 1994 (deck crew, 35-49y, males)	0.4318	0.2306	2.1%	1.54 [0.98, 2.42]
Brandt 1994 (deck crew, 50-64y, males)	0.4886	0.3322	1.7%	1.63 [0.85, 3.13]
Brandt 1994 (deck officers, 20-34y, males)	0.0198	0.2221	2.1%	1.02 [0.66, 1.58]
Brandt 1994 (deck officers, 35-49y, males)	0.3577	0.2776	1.9%	1.43 [0.83, 2.46]
Brandt 1994 (deck officers, 50-64y, males)	-0.9676	0.6811	0.8%	0.38 [0.10, 1.44]
Brandt 1994 (engine crew, 20-34y, males)	2.0281	0.5818	1.0%	7.60 [2.43, 23.77]
Brandt 1994 (engine crew, 35-49y, males)	1.4816	0.2684	1.9%	4.40 [2.60, 7.45]
Brandt 1994 (engine crew, 50-64y, males)	1.1787	0.1854	2.2%	3.25 [2.26, 4.67]
Brandt 1994 (engine officers, 20-34y, males)	0.8755	0.4782	1.2%	2.40 [0.94, 6.13]
Brandt 1994 (engine officers, 35-49y, males)	-0.3285	0.4137	1.4%	0.72 [0.32, 1.62]
Brandt 1994 (engine officers, 50-64y, males)	-0.462	0.6993	0.8%	0.63 [0.16, 2.48]
Guseva Canu 2019 (garbage collectors, males)	0.4187	0.2731	1.9%	1.52 [0.89, 2.60]
Guseva Canu 2019 (locomotive eng drivers, males)	-0.1863	0.1326	2.4%	0.83 [0.64, 1.08]
Guseva Canu 2019 (motor vehicle drivers, females)	0.6831	0.3384	1.7%	1.98 [1.02, 3.84]
Guseva Canu 2019 (motor vehicle drivers, males)	0.1484	0.0508	2.6%	1.16 [1.05, 1.28]
Guseva Canu 2019 (ship, air control&tech, males)	-0.0943	0.2477	2.0%	0.91 [0.56, 1.48]
Guseva Canu2019(transportclerks,recording,females)	0.0953	0.238	2.0%	1.10 [0.69, 1.75]
Guseva Canu2019(transport clerks,recording, males)	0.1398	0.0562	2.6%	1.15 [1.03, 1.28]
Hansen 1998 (seafarers, females)	1.7334	1.0737	0.4%	5.66 [0.69, 46.42]
Rafnsson 1993 (seamen, males)	0.3293	0.0836	2.5%	1.39 [1.18, 1.64]
Roberts 2013a(dockers and stevedores, all genders)	-0.1905	0.2092	2.1%	0.83 [0.55, 1.25]
Roberts 2013a (driving instructors, all genders)	-0.0827	0.2505	2.0%	0.92 [0.56, 1.50]
Roberts 2013a(forklift truck drivers, all genders)	-0.4112	0.1371	2.4%	0.66 [0.51, 0.87]
Roberts 2013a (merchant seafarers, all genders)	1.1398	0.0886	2.5%	3.13 [2.63, 3.72]
Roberts 2013a (rail operating staff, all genders)	-0.2893	0.1251	2.4%	0.75 [0.59, 0.96]
Roberts 2013b(dockers and stevedores, all genders)	0.3498	0.2587	2.0%	1.42 [0.85, 2.36]
Roberts 2013b (driving instructors, all genders)	-0.6938	0.2677	1.9%	0.50 [0.30, 0.84]
Roberts 2013b(forklift truck drivers, all genders)	0.2667	0.0954	2.5%	1.31 [1.08, 1.57]
Roberts 2013b (merchant seafarers, all genders)	1.1692	0.1722	2.3%	3.22 [2.30, 4.51]
Roberts 2013b (rail operating staff, all genders)	0.0851	0.23	2.1%	1.09 [0.69, 1.71]
Windsor-Shellard 2019 (forklift drivers, males)	0.4511	0.1163	2.4%	1.57 [1.25, 1.97]
**Subtotal (95% CI)**			**71.5%**	**1.39 [1.19, 1.63]**
Heterogeneity: Tau² = 0.17; Chi² = 300.83, df = 37 (P < 0.00001); I² = 88%				
Test for overall effect: Z = 4.10 (P < 0.0001)				
**1.4.4 Western Pacific**				
Andersen 2010 (general transport, all genders)	0.5878	0.0863	2.5%	1.80 [1.52, 2.13]
Too 2020 (mobile plant operators, all genders)	0.1875	0.0734	2.5%	1.21 [1.04, 1.39]
Too 2020 (road and rail drivers, all genders)	0.7303	0.0361	2.6%	2.08 [1.93, 2.23]
Wada 2016 (transport, machine operation, males)	0.4574	0.1236	2.4%	1.58 [1.24, 2.01]
Yamauchi 2018 (transport&postal, 30-39y, males)	0.2683	0.3272	1.7%	1.31 [0.69, 2.48]
Yamauchi 2018 (transport&postal, 40-49y, males)	-0.0339	0.3258	1.7%	0.97 [0.51, 1.83]
Yamauchi 2018 (transport&postal, 50-59y, males)	-0.499	0.4596	1.3%	0.61 [0.25, 1.49]
Yamauchi 2018 (transport&postal, over 60y, males)	-0.2231	0.7506	0.7%	0.80 [0.18, 3.48]
Yamauchi 2018 (transport&postal, under30y, males)	-0.1744	0.5927	1.0%	0.84 [0.26, 2.68]
**Subtotal (95% CI)**			**16.4%**	**1.40 [1.09, 1.79]**
Heterogeneity: Tau² = 0.08; Chi² = 59.38, df = 8 (P < 0.00001); I² = 87%				
Test for overall effect: Z = 2.67 (P = 0.008)				
**1.4.6 North America**				
Guidotti 1992 (bus drivers, males)	0.2647	0.358	1.6%	1.30 [0.65, 2.63]
Kposowa 1999 (transport equip operatives, all)	0.5878	0.4267	1.4%	1.80 [0.78, 4.15]
Mustard 2010 (motor transport, males)	0.3192	0.0938	2.5%	1.38 [1.14, 1.65]
Mustard 2010 (railway transport, males)	-0.3696	0.4139	1.4%	0.69 [0.31, 1.56]
Stallones 2013 (transport,material moving,females)	-0.5225	0.0571	2.6%	0.59 [0.53, 0.66]
Stallones 2013 (transport, material moving, males)	-0.1507	0.0257	2.6%	0.86 [0.82, 0.90]
**Subtotal (95% CI)**			**12.1%**	**0.95 [0.71, 1.28]**
Heterogeneity: Tau² = 0.09; Chi² = 70.59, df = 5 (P < 0.00001); I² = 93%				
Test for overall effect: Z = 0.31 (P = 0.76)				
**Total (95% CI)**			**100.0%**	**1.32 [1.15, 1.52]**
Heterogeneity: Tau² = 0.20; Chi² = 901.02, df = 52 (P < 0.00001); I² = 94%				
Test for overall effect: Z = 3.83 (P = 0.0001)				
Test for subgroup differences: Chi² = 5.22, df = 2 (P = 0.07), I² = 61.7%				

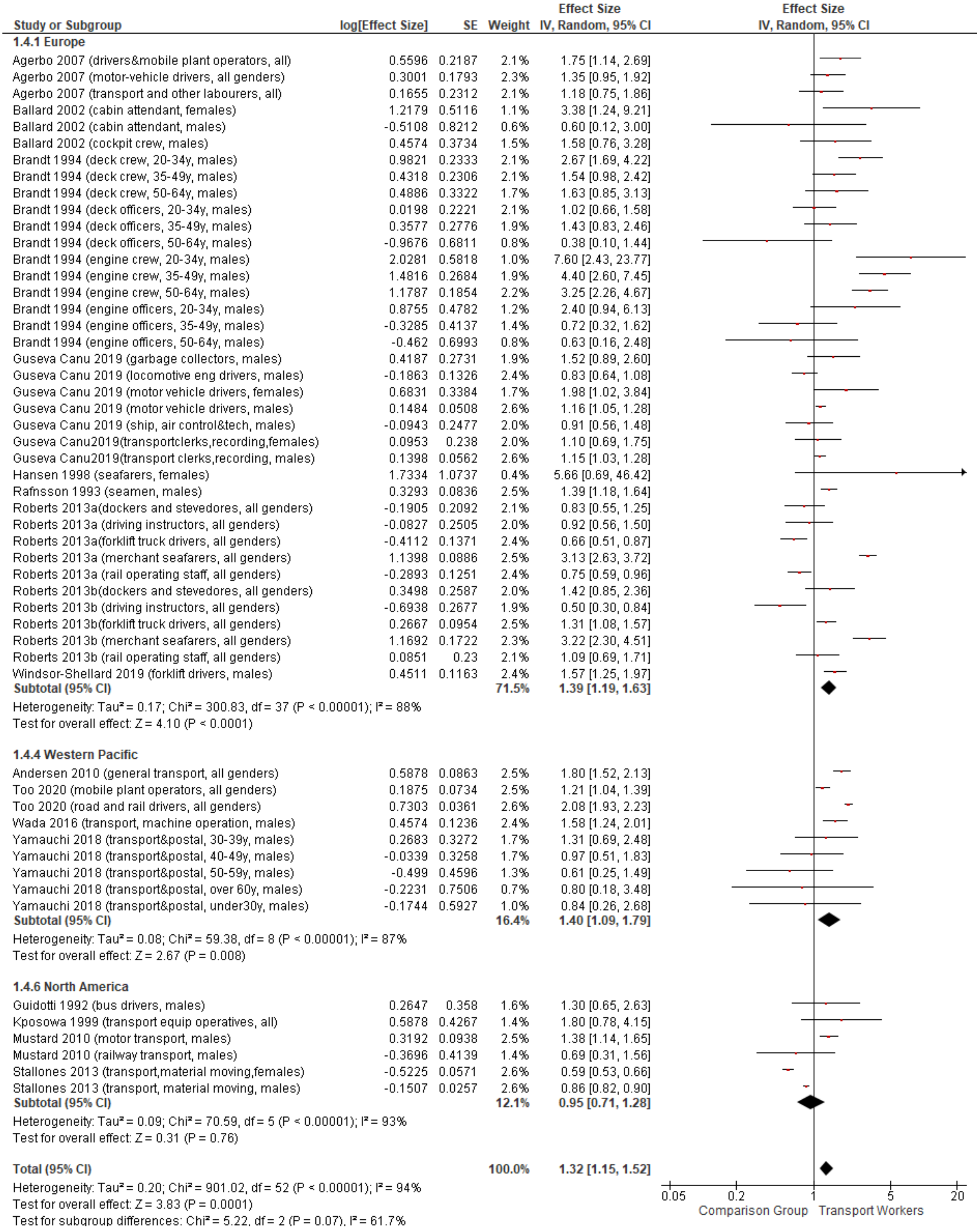

D. By Data Collection Period

Study or Subgroup	log[Effect Size]	SE	Weight	Effect Size IV, Random, 95% CI
**1.5.1 Data Collected From 1989 and before**				
Brandt 1994 (deck crew, 20-34y, males)	0.9821	0.2333	2.1%	2.67 [1.69, 4.22]
Brandt 1994 (deck crew, 35-49y, males)	0.4318	0.2306	2.1%	1.54 [0.98, 2.42]
Brandt 1994 (deck crew, 50-64y, males)	0.4886	0.3322	1.7%	1.63 [0.85, 3.13]
Brandt 1994 (deck officers, 20-34y, males)	0.0198	0.2221	2.1%	1.02 [0.66, 1.58]
Brandt 1994 (deck officers, 35-49y, males)	0.3577	0.2776	1.9%	1.43 [0.83, 2.46]
Brandt 1994 (deck officers, 50-64y, males)	-0.9676	0.6811	0.8%	0.38 [0.10, 1.44]
Brandt 1994 (engine crew, 20-34y, males)	2.0281	0.5818	1.0%	7.60 [2.43, 23.77]
Brandt 1994 (engine crew, 35-49y, males)	1.4816	0.2684	1.9%	4.40 [2.60, 7.45]
Brandt 1994 (engine crew, 50-64y, males)	1.1787	0.1854	2.2%	3.25 [2.26, 4.67]
Brandt 1994 (engine officers, 20-34y, males)	0.8755	0.4782	1.2%	2.40 [0.94, 6.13]
Brandt 1994 (engine officers, 35-49y, males)	-0.3285	0.4137	1.4%	0.72 [0.32, 1.62]
Brandt 1994 (engine officers, 50-64y, males)	-0.462	0.6993	0.8%	0.63 [0.16, 2.48]
Guidotti 1992 (bus drivers, males)	0.2647	0.358	1.6%	1.30 [0.65, 2.63]
Kposowa 1999 (transport equip operatives, all)	0.5878	0.4267	1.4%	1.80 [0.78, 4.15]
Rafnsson 1993 (seamen, males)	0.3293	0.0836	2.5%	1.39 [1.18, 1.64]
Roberts 2013a(dockers and stevedores, all genders)	-0.1905	0.2092	2.1%	0.83 [0.55, 1.25]
Roberts 2013a (driving instructors, all genders)	-0.0827	0.2505	2.0%	0.92 [0.56, 1.50]
Roberts 2013a(forklift truck drivers, all genders)	-0.4112	0.1371	2.4%	0.66 [0.51, 0.87]
Roberts 2013a (merchant seafarers, all genders)	1.1398	0.0886	2.5%	3.13 [2.63, 3.72]
Roberts 2013a (rail operating staff, all genders)	-0.2893	0.1251	2.4%	0.75 [0.59, 0.96]
**Subtotal (95% CI)**			**36.1%**	**1.48 [1.09, 2.00]**
Heterogeneity: Tau² = 0.38; Chi² = 208.05, df = 19 (P < 0.00001); I² = 91%				
Test for overall effect: Z = 2.51 (P = 0.01)				
**1.5.2 Data Collected From 1990-2004**				
Agerbo 2007 (drivers&mobile plant operators, all)	0.5596	0.2187	2.1%	1.75 [1.14, 2.69]
Agerbo 2007 (motor-vehicle drivers, all genders)	0.3001	0.1793	2.3%	1.35 [0.95, 1.92]
Agerbo 2007 (transport and other labourers, all)	0.1655	0.2312	2.1%	1.18 [0.75, 1.86]
Ballard 2002 (cabin attendant, females)	1.2179	0.5116	1.1%	3.38 [1.24, 9.21]
Ballard 2002 (cabin attendant, males)	-0.5108	0.8212	0.6%	0.60 [0.12, 3.00]
Ballard 2002 (cockpit crew, males)	0.4574	0.3734	1.5%	1.58 [0.76, 3.28]
Hansen 1998 (seafarers, females)	1.7334	1.0737	0.4%	5.66 [0.69, 46.42]
Mustard 2010 (motor transport, males)	0.3192	0.0938	2.5%	1.38 [1.14, 1.65]
Mustard 2010 (railway transport, males)	-0.3696	0.4139	1.4%	0.69 [0.31, 1.56]
**Subtotal (95% CI)**			**14.0%**	**1.40 [1.15, 1.69]**
Heterogeneity: Tau² = 0.02; Chi² = 10.39, df = 8 (P = 0.24); I² = 23%				
Test for overall effect: Z = 3.40 (P = 0.0007)				
**1.5.3 Data Collected From 2005 onwards**				
Andersen 2010 (general transport, all genders)	0.5878	0.0863	2.5%	1.80 [1.52, 2.13]
Guseva Canu 2019 (garbage collectors, males)	0.4187	0.2731	1.9%	1.52 [0.89, 2.60]
Guseva Canu 2019 (locomotive eng drivers, males)	-0.1863	0.1326	2.4%	0.83 [0.64, 1.08]
Guseva Canu 2019 (motor vehicle drivers, females)	0.6831	0.3384	1.7%	1.98 [1.02, 3.84]
Guseva Canu 2019 (motor vehicle drivers, males)	0.1484	0.0508	2.6%	1.16 [1.05, 1.28]
Guseva Canu 2019 (ship, air control&tech, males)	-0.0943	0.2477	2.0%	0.91 [0.56, 1.48]
Guseva Canu2019(transportclerks,recording,females)	0.0953	0.238	2.0%	1.10 [0.69, 1.75]
Guseva Canu2019(transport clerks,recording, males)	0.1398	0.0562	2.6%	1.15 [1.03, 1.28]
Roberts 2013b(dockers and stevedores, all genders)	0.3498	0.2587	2.0%	1.42 [0.85, 2.36]
Roberts 2013b (driving instructors, all genders)	-0.6938	0.2677	1.9%	0.50 [0.30, 0.84]
Roberts 2013b(forklift truck drivers, all genders)	0.2667	0.0954	2.5%	1.31 [1.08, 1.57]
Roberts 2013b (merchant seafarers, all genders)	1.1692	0.1722	2.3%	3.22 [2.30, 4.51]
Roberts 2013b (rail operating staff, all genders)	0.0851	0.23	2.1%	1.09 [0.69, 1.71]
Stallones 2013 (transport,material moving,females)	-0.5225	0.0571	2.6%	0.59 [0.53, 0.66]
Stallones 2013 (transport, material moving, males)	-0.1507	0.0257	2.6%	0.86 [0.82, 0.90]
Too 2020 (mobile plant operators, all genders)	0.1875	0.0734	2.5%	1.21 [1.04, 1.39]
Too 2020 (road and rail drivers, all genders)	0.7303	0.0361	2.6%	2.08 [1.93, 2.23]
Wada 2016 (transport, machine operation, males)	0.4574	0.1236	2.4%	1.58 [1.24, 2.01]
Windsor-Shellard 2019 (forklift drivers, males)	0.4511	0.1163	2.4%	1.57 [1.25, 1.97]
Yamauchi 2018 (transport&postal, 30-39y, males)	0.2683	0.3272	1.7%	1.31 [0.69, 2.48]
Yamauchi 2018 (transport&postal, 40-49y, males)	-0.0339	0.3258	1.7%	0.97 [0.51, 1.83]
Yamauchi 2018 (transport&postal, 50-59y, males)	-0.499	0.4596	1.3%	0.61 [0.25, 1.49]
Yamauchi 2018 (transport&postal, over 60y, males)	-0.2231	0.7506	0.7%	0.80 [0.18, 3.48]
Yamauchi 2018 (transport&postal, under30y, males)	-0.1744	0.5927	1.0%	0.84 [0.26, 2.68]
**Subtotal (95% CI)**			**49.9%**	**1.20 [0.98, 1.45]**
Heterogeneity: Tau² = 0.19; Chi² = 631.71, df = 23 (P < 0.00001); I² = 96%				
Test for overall effect: Z = 1.80 (P = 0.07)				
**Total (95% CI)**			**100.0%**	**1.32 [1.15, 1.52]**
Heterogeneity: Tau² = 0.20; Chi² = 901.02, df = 52 (P < 0.00001); I² = 94%				
Test for overall effect: Z = 3.83 (P = 0.0001)				
Test for subgroup differences: Chi² = 1.84, df = 2 (P = 0.40), I² = 0%				

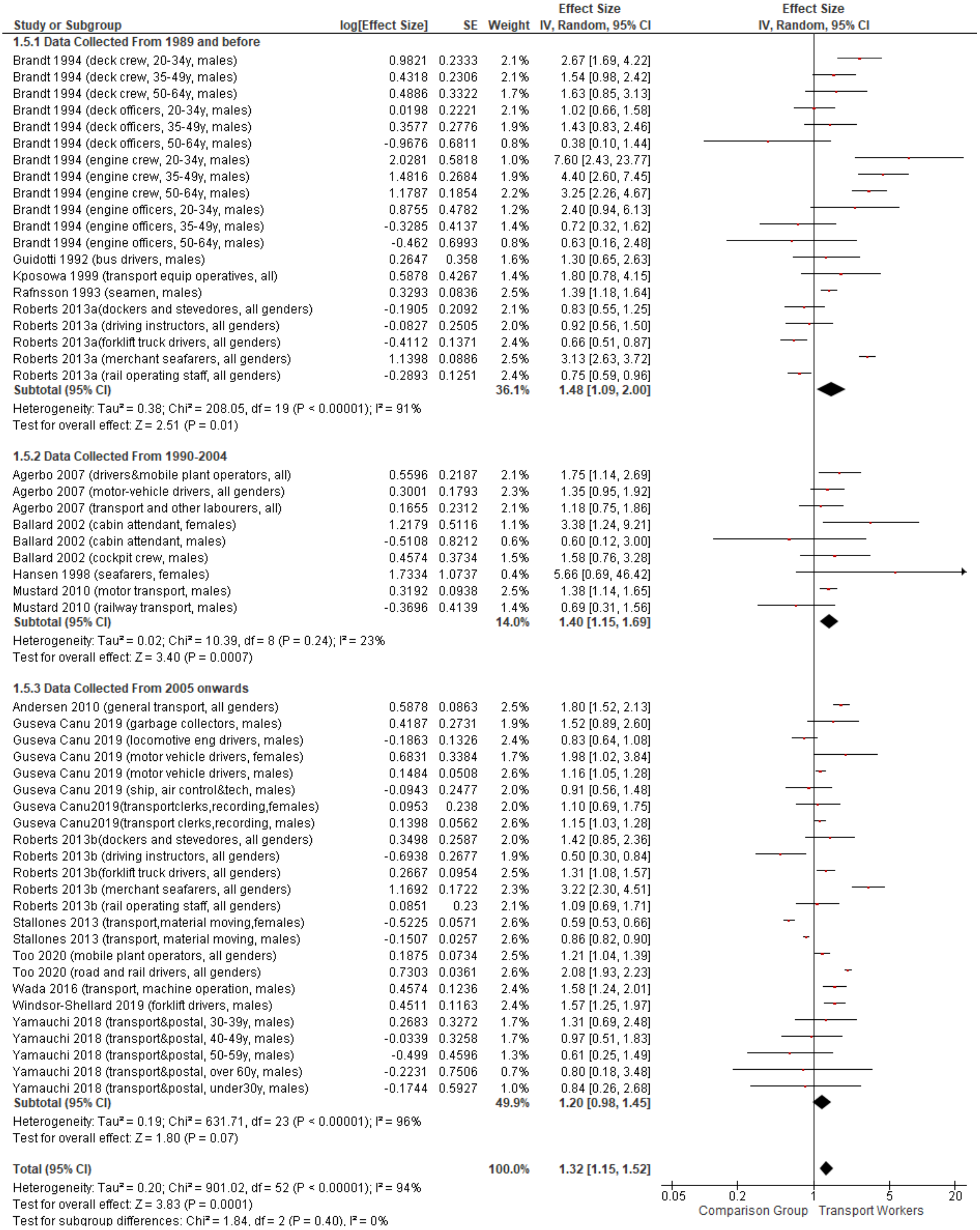

E. By Comparison Group

Study or Subgroup	log[Effect Size]	SE	Weight	Effect Size IV, Random, 95% CI
**1.6.1 General Population**				
Ballard 2002 (cabin attendant, females)	1.2179	0.5116	1.1%	3.38 [1.24, 9.21]
Ballard 2002 (cabin attendant, males)	-0.5108	0.8212	0.6%	0.60 [0.12, 3.00]
Ballard 2002 (cockpit crew, males)	0.4574	0.3734	1.5%	1.58 [0.76, 3.28]
Guidotti 1992 (bus drivers, males)	0.2647	0.358	1.6%	1.30 [0.65, 2.63]
Guseva Canu 2019 (garbage collectors, males)	0.4187	0.2731	1.9%	1.52 [0.89, 2.60]
Guseva Canu 2019 (locomotive eng drivers, males)	-0.1863	0.1326	2.4%	0.83 [0.64, 1.08]
Guseva Canu 2019 (motor vehicle drivers, females)	0.6831	0.3384	1.7%	1.98 [1.02, 3.84]
Guseva Canu 2019 (motor vehicle drivers, males)	0.1484	0.0508	2.6%	1.16 [1.05, 1.28]
Guseva Canu 2019 (ship, air control&tech, males)	-0.0943	0.2477	2.0%	0.91 [0.56, 1.48]
Guseva Canu2019(transportclerks,recording,females)	0.0953	0.238	2.0%	1.10 [0.69, 1.75]
Guseva Canu2019(transport clerks,recording, males)	0.1398	0.0562	2.6%	1.15 [1.03, 1.28]
Hansen 1998 (seafarers, females)	1.7334	1.0737	0.4%	5.66 [0.69, 46.42]
Rafnsson 1993 (seamen, males)	0.3293	0.0836	2.5%	1.39 [1.18, 1.64]
Windsor-Shellard 2019 (forklift drivers, males)	0.4511	0.1163	2.4%	1.57 [1.25, 1.97]
**Subtotal (95% CI)**			**25.4%**	**1.24 [1.09, 1.41]**
Heterogeneity: Tau² = 0.02; Chi² = 28.99, df = 13 (P = 0.007); I² = 55%				
Test for overall effect: Z = 3.36 (P = 0.0008)				
**1.6.2 Employed Population**				
Andersen 2010 (general transport, all genders)	0.5878	0.0863	2.5%	1.80 [1.52, 2.13]
Brandt 1994 (deck crew, 20-34y, males)	0.9821	0.2333	2.1%	2.67 [1.69, 4.22]
Brandt 1994 (deck crew, 35-49y, males)	0.4318	0.2306	2.1%	1.54 [0.98, 2.42]
Brandt 1994 (deck crew, 50-64y, males)	0.4886	0.3322	1.7%	1.63 [0.85, 3.13]
Brandt 1994 (deck officers, 20-34y, males)	0.0198	0.2221	2.1%	1.02 [0.66, 1.58]
Brandt 1994 (deck officers, 35-49y, males)	0.3577	0.2776	1.9%	1.43 [0.83, 2.46]
Brandt 1994 (deck officers, 50-64y, males)	-0.9676	0.6811	0.8%	0.38 [0.10, 1.44]
Brandt 1994 (engine crew, 20-34y, males)	2.0281	0.5818	1.0%	7.60 [2.43, 23.77]
Brandt 1994 (engine crew, 35-49y, males)	1.4816	0.2684	1.9%	4.40 [2.60, 7.45]
Brandt 1994 (engine crew, 50-64y, males)	1.1787	0.1854	2.2%	3.25 [2.26, 4.67]
Brandt 1994 (engine officers, 20-34y, males)	0.8755	0.4782	1.2%	2.40 [0.94, 6.13]
Brandt 1994 (engine officers, 35-49y, males)	-0.3285	0.4137	1.4%	0.72 [0.32, 1.62]
Brandt 1994 (engine officers, 50-64y, males)	-0.462	0.6993	0.8%	0.63 [0.16, 2.48]
Mustard 2010 (motor transport, males)	0.3192	0.0938	2.5%	1.38 [1.14, 1.65]
Mustard 2010 (railway transport, males)	-0.3696	0.4139	1.4%	0.69 [0.31, 1.56]
Stallones 2013 (transport,material moving,females)	-0.5225	0.0571	2.6%	0.59 [0.53, 0.66]
Stallones 2013 (transport, material moving, males)	-0.1507	0.0257	2.6%	0.86 [0.82, 0.90]
Too 2020 (mobile plant operators, all genders)	0.1875	0.0734	2.5%	1.21 [1.04, 1.39]
Too 2020 (road and rail drivers, all genders)	0.7303	0.0361	2.6%	2.08 [1.93, 2.23]
**Subtotal (95% CI)**			**35.9%**	**1.47 [1.12, 1.93]**
Heterogeneity: Tau² = 0.29; Chi² = 652.78, df = 18 (P < 0.00001); I² = 97%				
Test for overall effect: Z = 2.75 (P = 0.006)				
**1.6.3 Selected occupations**				
Roberts 2013a(dockers and stevedores, all genders)	-0.1905	0.2092	2.1%	0.83 [0.55, 1.25]
Roberts 2013a (driving instructors, all genders)	-0.0827	0.2505	2.0%	0.92 [0.56, 1.50]
Roberts 2013a(forklift truck drivers, all genders)	-0.4112	0.1371	2.4%	0.66 [0.51, 0.87]
Roberts 2013a (merchant seafarers, all genders)	1.1398	0.0886	2.5%	3.13 [2.63, 3.72]
Roberts 2013a (rail operating staff, all genders)	-0.2893	0.1251	2.4%	0.75 [0.59, 0.96]
Roberts 2013b(dockers and stevedores, all genders)	0.3498	0.2587	2.0%	1.42 [0.85, 2.36]
Roberts 2013b (driving instructors, all genders)	-0.6938	0.2677	1.9%	0.50 [0.30, 0.84]
Roberts 2013b(forklift truck drivers, all genders)	0.2667	0.0954	2.5%	1.31 [1.08, 1.57]
Roberts 2013b (merchant seafarers, all genders)	1.1692	0.1722	2.3%	3.22 [2.30, 4.51]
Roberts 2013b (rail operating staff, all genders)	0.0851	0.23	2.1%	1.09 [0.69, 1.71]
Yamauchi 2018 (transport&postal, 30-39y, males)	0.2683	0.3272	1.7%	1.31 [0.69, 2.48]
Yamauchi 2018 (transport&postal, 40-49y, males)	-0.0339	0.3258	1.7%	0.97 [0.51, 1.83]
Yamauchi 2018 (transport&postal, 50-59y, males)	-0.499	0.4596	1.3%	0.61 [0.25, 1.49]
Yamauchi 2018 (transport&postal, over 60y, males)	-0.2231	0.7506	0.7%	0.80 [0.18, 3.48]
Yamauchi 2018 (transport&postal, under30y, males)	-0.1744	0.5927	1.0%	0.84 [0.26, 2.68]
**Subtotal (95% CI)**			**28.5%**	**1.08 [0.76, 1.55]**
Heterogeneity: Tau² = 0.42; Chi² = 191.93, df = 14 (P < 0.00001); I² = 93%				
Test for overall effect: Z = 0.44 (P = 0.66)				
**1.6.4 One specific occupation**				
Agerbo 2007 (drivers&mobile plant operators, all)	0.5596	0.2187	2.1%	1.75 [1.14, 2.69]
Agerbo 2007 (motor-vehicle drivers, all genders)	0.3001	0.1793	2.3%	1.35 [0.95, 1.92]
Agerbo 2007 (transport and other labourers, all)	0.1655	0.2312	2.1%	1.18 [0.75, 1.86]
Kposowa 1999 (transport equip operatives, all)	0.5878	0.4267	1.4%	1.80 [0.78, 4.15]
Wada 2016 (transport, machine operation, males)	0.4574	0.1236	2.4%	1.58 [1.24, 2.01]
**Subtotal (95% CI)**			**10.2%**	**1.50 [1.27, 1.77]**
Heterogeneity: Tau² = 0.00; Chi² = 2.28, df = 4 (P = 0.68); I² = 0%				
Test for overall effect: Z = 4.81 (P < 0.00001)				
**Total (95% CI)**			**100.0%**	**1.32 [1.15, 1.52]**
Heterogeneity: Tau² = 0.20; Chi² = 901.02, df = 52 (P < 0.00001); I² = 94%				
Test for overall effect: Z = 3.83 (P = 0.0001)				
Test for subgroup differences: Chi² = 4.90, df = 3 (P = 0.18), I² = 38.8%				

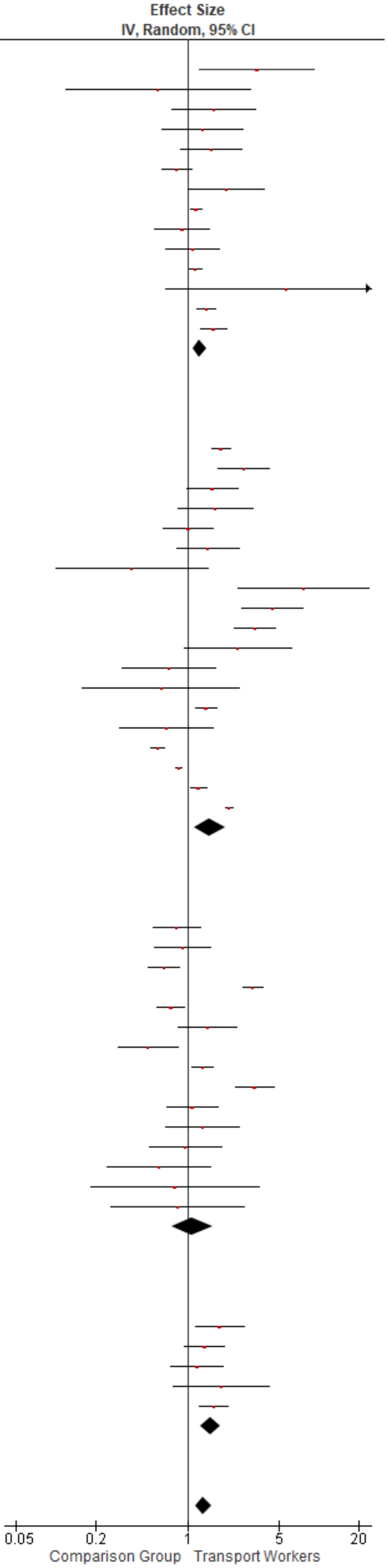

F. By Effect Measure

Study or Subgroup	log[Effect Size]	SE	Weight	Effect Size IV, Random, 95% CI
**1.7.1 Rate Ratio**				
Agerbo 2007 (drivers&mobile plant operators, all)	0.5596	0.2187	2.1%	1.75 [1.14, 2.69]
Agerbo 2007 (motor-vehicle drivers, all genders)	0.3001	0.1793	2.3%	1.35 [0.95, 1.92]
Agerbo 2007 (transport and other labourers, all)	0.1655	0.2312	2.1%	1.18 [0.75, 1.86]
Andersen 2010 (general transport, all genders)	0.5878	0.0863	2.5%	1.80 [1.52, 2.13]
Brandt 1994 (deck crew, 20-34y, males)	0.9821	0.2333	2.1%	2.67 [1.69, 4.22]
Brandt 1994 (deck crew, 35-49y, males)	0.4318	0.2306	2.1%	1.54 [0.98, 2.42]
Brandt 1994 (deck crew, 50-64y, males)	0.4886	0.3322	1.7%	1.63 [0.85, 3.13]
Brandt 1994 (deck officers, 20-34y, males)	0.0198	0.2221	2.1%	1.02 [0.66, 1.58]
Brandt 1994 (deck officers, 35-49y, males)	0.3577	0.2776	1.9%	1.43 [0.83, 2.46]
Brandt 1994 (deck officers, 50-64y, males)	-0.9676	0.6811	0.8%	0.38 [0.10, 1.44]
Brandt 1994 (engine crew, 20-34y, males)	2.0281	0.5818	1.0%	7.60 [2.43, 23.77]
Brandt 1994 (engine crew, 35-49y, males)	1.4816	0.2684	1.9%	4.40 [2.60, 7.45]
Brandt 1994 (engine crew, 50-64y, males)	1.1787	0.1854	2.2%	3.25 [2.26, 4.67]
Brandt 1994 (engine officers, 20-34y, males)	0.8755	0.4782	1.2%	2.40 [0.94, 6.13]
Brandt 1994 (engine officers, 35-49y, males)	-0.3285	0.4137	1.4%	0.72 [0.32, 1.62]
Brandt 1994 (engine officers, 50-64y, males)	-0.462	0.6993	0.8%	0.63 [0.16, 2.48]
Kposowa 1999 (transport equip operatives, all)	0.5878	0.4267	1.4%	1.80 [0.78, 4.15]
Mustard 2010 (motor transport, males)	0.3192	0.0938	2.5%	1.38 [1.14, 1.65]
Mustard 2010 (railway transport, males)	-0.3696	0.4139	1.4%	0.69 [0.31, 1.56]
Roberts 2013a(dockers and stevedores, all genders)	-0.1905	0.2092	2.1%	0.83 [0.55, 1.25]
Roberts 2013a (driving instructors, all genders)	-0.0827	0.2505	2.0%	0.92 [0.56, 1.50]
Roberts 2013a(forklift truck drivers, all genders)	-0.4112	0.1371	2.4%	0.66 [0.51, 0.87]
Roberts 2013a (merchant seafarers, all genders)	1.1398	0.0886	2.5%	3.13 [2.63, 3.72]
Roberts 2013a (rail operating staff, all genders)	-0.2893	0.1251	2.4%	0.75 [0.59, 0.96]
Roberts 2013b(dockers and stevedores, all genders)	0.3498	0.2587	2.0%	1.42 [0.85, 2.36]
Roberts 2013b (driving instructors, all genders)	-0.6938	0.2677	1.9%	0.50 [0.30, 0.84]
Roberts 2013b(forklift truck drivers, all genders)	0.2667	0.0954	2.5%	1.31 [1.08, 1.57]
Roberts 2013b (merchant seafarers, all genders)	1.1692	0.1722	2.3%	3.22 [2.30, 4.51]
Roberts 2013b (rail operating staff, all genders)	0.0851	0.23	2.1%	1.09 [0.69, 1.71]
Stallones 2013 (transport,material moving,females)	-0.5225	0.0571	2.6%	0.59 [0.53, 0.66]
Stallones 2013 (transport, material moving, males)	-0.1507	0.0257	2.6%	0.86 [0.82, 0.90]
Too 2020 (mobile plant operators, all genders)	0.1875	0.0734	2.5%	1.21 [1.04, 1.39]
Too 2020 (road and rail drivers, all genders)	0.7303	0.0361	2.6%	2.08 [1.93, 2.23]
Wada 2016 (transport, machine operation, males)	0.4574	0.1236	2.4%	1.58 [1.24, 2.01]
Yamauchi 2018 (transport&postal, 30-39y, males)	0.2683	0.3272	1.7%	1.31 [0.69, 2.48]
Yamauchi 2018 (transport&postal, 40-49y, males)	-0.0339	0.3258	1.7%	0.97 [0.51, 1.83]
Yamauchi 2018 (transport&postal, 50-59y, males)	-0.499	0.4596	1.3%	0.61 [0.25, 1.49]
Yamauchi 2018 (transport&postal, over 60y, males)	-0.2231	0.7506	0.7%	0.80 [0.18, 3.48]
Yamauchi 2018 (transport&postal, under30y, males)	-0.1744	0.5927	1.0%	0.84 [0.26, 2.68]
**Subtotal (95% CI)**			**74.6%**	**1.32 [1.09, 1.59]**
Heterogeneity: Tau² = 0.28; Chi² = 871.80, df = 38 (P < 0.00001); I² = 96%				
Test for overall effect: Z = 2.88 (P = 0.004)				
**1.7.2 SMR**				
Ballard 2002 (cabin attendant, females)	1.2179	0.5116	1.1%	3.38 [1.24, 9.21]
Ballard 2002 (cabin attendant, males)	-0.5108	0.8212	0.6%	0.60 [0.12, 3.00]
Ballard 2002 (cockpit crew, males)	0.4574	0.3734	1.5%	1.58 [0.76, 3.28]
Guseva Canu 2019 (garbage collectors, males)	0.4187	0.2731	1.9%	1.52 [0.89, 2.60]
Guseva Canu 2019 (locomotive eng drivers, males)	-0.1863	0.1326	2.4%	0.83 [0.64, 1.08]
Guseva Canu 2019 (motor vehicle drivers, females)	0.6831	0.3384	1.7%	1.98 [1.02, 3.84]
Guseva Canu 2019 (motor vehicle drivers, males)	0.1484	0.0508	2.6%	1.16 [1.05, 1.28]
Guseva Canu 2019 (ship, air control&tech, males)	-0.0943	0.2477	2.0%	0.91 [0.56, 1.48]
Guseva Canu2019(transportclerks,recording,females)	0.0953	0.238	2.0%	1.10 [0.69, 1.75]
Guseva Canu2019(transport clerks,recording, males)	0.1398	0.0562	2.6%	1.15 [1.03, 1.28]
Hansen 1998 (seafarers, females)	1.7334	1.0737	0.4%	5.66 [0.69, 46.42]
Rafnsson 1993 (seamen, males)	0.3293	0.0836	2.5%	1.39 [1.18, 1.64]
Windsor-Shellard 2019 (forklift drivers, males)	0.4511	0.1163	2.4%	1.57 [1.25, 1.97]
**Subtotal (95% CI)**			**23.8%**	**1.24 [1.09, 1.42]**
Heterogeneity: Tau² = 0.02; Chi² = 28.94, df = 12 (P = 0.004); I² = 59%				
Test for overall effect: Z = 3.24 (P = 0.001)				
**1.7.3 PMR**				
Guidotti 1992 (bus drivers, males)	0.2647	0.358	1.6%	1.30 [0.65, 2.63]
**Subtotal (95% CI)**			**1.6%**	**1.30 [0.65, 2.63]**
Heterogeneity: Not applicable				
Test for overall effect: Z = 0.74 (P = 0.46)				
**Total (95% CI)**			**100.0%**	**1.32 [1.15, 1.52]**
Heterogeneity: Tau² = 0.20; Chi² = 901.02, df = 52 (P < 0.00001); I² = 94%				
Test for overall effect: Z = 3.83 (P = 0.0001)				
Test for subgroup differences: Chi² = 0.26, df = 2 (P = 0.88), I² = 0%				

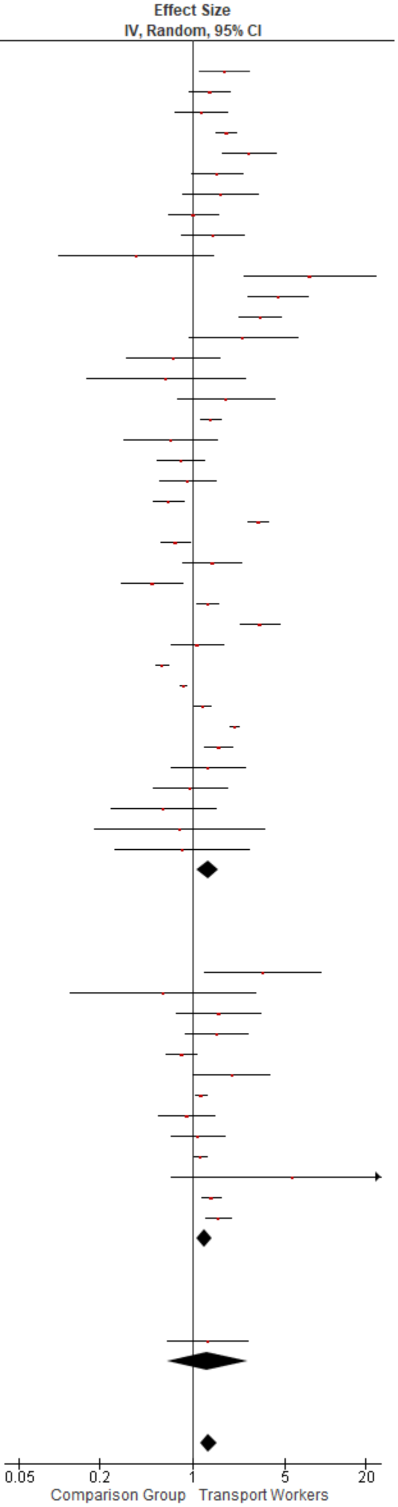

G. By Study Design

Study or Subgroup	log[Effect Size]	SE	Weight	Effect Size IV, Random, 95% CI
**1.8.1 Case-series**				
Andersen 2010 (general transport, all genders)	0.5878	0.0863	2.5%	1.80 [1.52, 2.13]
Roberts 2013a(dockers and stevedores, all genders)	-0.1905	0.2092	2.1%	0.83 [0.55, 1.25]
Roberts 2013a (driving instructors, all genders)	-0.0827	0.2505	2.0%	0.92 [0.56, 1.50]
Roberts 2013a(forklift truck drivers, all genders)	-0.4112	0.1371	2.4%	0.66 [0.51, 0.87]
Roberts 2013a (merchant seafarers, all genders)	1.1398	0.0886	2.5%	3.13 [2.63, 3.72]
Roberts 2013a (rail operating staff, all genders)	-0.2893	0.1251	2.4%	0.75 [0.59, 0.96]
Roberts 2013b(dockers and stevedores, all genders)	0.3498	0.2587	2.0%	1.42 [0.85, 2.36]
Roberts 2013b (driving instructors, all genders)	-0.6938	0.2677	1.9%	0.50 [0.30, 0.84]
Roberts 2013b(forklift truck drivers, all genders)	0.2667	0.0954	2.5%	1.31 [1.08, 1.57]
Roberts 2013b (merchant seafarers, all genders)	1.1692	0.1722	2.3%	3.22 [2.30, 4.51]
Roberts 2013b (rail operating staff, all genders)	0.0851	0.23	2.1%	1.09 [0.69, 1.71]
Stallones 2013 (transport,material moving,females)	-0.5225	0.0571	2.6%	0.59 [0.53, 0.66]
Stallones 2013 (transport, material moving, males)	-0.1507	0.0257	2.6%	0.86 [0.82, 0.90]
Wada 2016 (transport, machine operation, males)	0.4574	0.1236	2.4%	1.58 [1.24, 2.01]
Windsor-Shellard 2019 (forklift drivers, males)	0.4511	0.1163	2.4%	1.57 [1.25, 1.97]
Yamauchi 2018 (transport&postal, 30-39y, males)	0.2683	0.3272	1.7%	1.31 [0.69, 2.48]
Yamauchi 2018 (transport&postal, 40-49y, males)	-0.0339	0.3258	1.7%	0.97 [0.51, 1.83]
Yamauchi 2018 (transport&postal, 50-59y, males)	-0.499	0.4596	1.3%	0.61 [0.25, 1.49]
Yamauchi 2018 (transport&postal, over 60y, males)	-0.2231	0.7506	0.7%	0.80 [0.18, 3.48]
Yamauchi 2018 (transport&postal, under30y, males)	-0.1744	0.5927	1.0%	0.84 [0.26, 2.68]
**Subtotal (95% CI)**			**41.1%**	**1.12 [0.88, 1.43]**
Heterogeneity: Tau² = 0.25; Chi² = 436.06, df = 19 (P < 0.00001); I² = 96%				
Test for overall effect: Z = 0.91 (P = 0.36)				
**1.8.2 Prospective Cohort**				
Guseva Canu 2019 (garbage collectors, males)	0.4187	0.2731	1.9%	1.52 [0.89, 2.60]
Guseva Canu 2019 (locomotive eng drivers, males)	-0.1863	0.1326	2.4%	0.83 [0.64, 1.08]
Guseva Canu 2019 (motor vehicle drivers, females)	0.6831	0.3384	1.7%	1.98 [1.02, 3.84]
Guseva Canu 2019 (motor vehicle drivers, males)	0.1484	0.0508	2.6%	1.16 [1.05, 1.28]
Guseva Canu 2019 (ship, air control&tech, males)	-0.0943	0.2477	2.0%	0.91 [0.56, 1.48]
Guseva Canu2019(transportclerks,recording,females)	0.0953	0.238	2.0%	1.10 [0.69, 1.75]
Guseva Canu2019(transport clerks,recording, males)	0.1398	0.0562	2.6%	1.15 [1.03, 1.28]
Kposowa 1999 (transport equip operatives, all)	0.5878	0.4267	1.4%	1.80 [0.78, 4.15]
Mustard 2010 (motor transport, males)	0.3192	0.0938	2.5%	1.38 [1.14, 1.65]
Mustard 2010 (railway transport, males)	-0.3696	0.4139	1.4%	0.69 [0.31, 1.56]
**Subtotal (95% CI)**			**20.4%**	**1.16 [1.03, 1.30]**
Heterogeneity: Tau² = 0.01; Chi² = 16.82, df = 9 (P = 0.05); I² = 47%				
Test for overall effect: Z = 2.41 (P = 0.02)				
**1.8.3 Retrospective cohort**				
Ballard 2002 (cabin attendant, females)	1.2179	0.5116	1.1%	3.38 [1.24, 9.21]
Ballard 2002 (cabin attendant, males)	-0.5108	0.8212	0.6%	0.60 [0.12, 3.00]
Ballard 2002 (cockpit crew, males)	0.4574	0.3734	1.5%	1.58 [0.76, 3.28]
Brandt 1994 (deck crew, 20-34y, males)	0.9821	0.2333	2.1%	2.67 [1.69, 4.22]
Brandt 1994 (deck crew, 35-49y, males)	0.4318	0.2306	2.1%	1.54 [0.98, 2.42]
Brandt 1994 (deck crew, 50-64y, males)	0.4886	0.3322	1.7%	1.63 [0.85, 3.13]
Brandt 1994 (deck officers, 20-34y, males)	0.0198	0.2221	2.1%	1.02 [0.66, 1.58]
Brandt 1994 (deck officers, 35-49y, males)	0.3577	0.2776	1.9%	1.43 [0.83, 2.46]
Brandt 1994 (deck officers, 50-64y, males)	-0.9676	0.6811	0.8%	0.38 [0.10, 1.44]
Brandt 1994 (engine crew, 20-34y, males)	2.0281	0.5818	1.0%	7.60 [2.43, 23.77]
Brandt 1994 (engine crew, 35-49y, males)	1.4816	0.2684	1.9%	4.40 [2.60, 7.45]
Brandt 1994 (engine crew, 50-64y, males)	1.1787	0.1854	2.2%	3.25 [2.26, 4.67]
Brandt 1994 (engine officers, 20-34y, males)	0.8755	0.4782	1.2%	2.40 [0.94, 6.13]
Brandt 1994 (engine officers, 35-49y, males)	-0.3285	0.4137	1.4%	0.72 [0.32, 1.62]
Brandt 1994 (engine officers, 50-64y, males)	-0.462	0.6993	0.8%	0.63 [0.16, 2.48]
Guidotti 1992 (bus drivers, males)	0.2647	0.358	1.6%	1.30 [0.65, 2.63]
Hansen 1998 (seafarers, females)	1.7334	1.0737	0.4%	5.66 [0.69, 46.42]
Rafnsson 1993 (seamen, males)	0.3293	0.0836	2.5%	1.39 [1.18, 1.64]
**Subtotal (95% CI)**			**26.9%**	**1.75 [1.33, 2.31]**
Heterogeneity: Tau² = 0.20; Chi² = 63.55, df = 17 (P < 0.00001); I² = 73%				
Test for overall effect: Z = 4.00 (P < 0.0001)				
**1.8.4 Nested case-control**				
Agerbo 2007 (drivers&mobile plant operators, all)	0.5596	0.2187	2.1%	1.75 [1.14, 2.69]
Agerbo 2007 (motor-vehicle drivers, all genders)	0.3001	0.1793	2.3%	1.35 [0.95, 1.92]
Agerbo 2007 (transport and other labourers, all)	0.1655	0.2312	2.1%	1.18 [0.75, 1.86]
**Subtotal (95% CI)**			**6.4%**	**1.41 [1.11, 1.78]**
Heterogeneity: Tau² = 0.00; Chi² = 1.63, df = 2 (P = 0.44); I² = 0%				
Test for overall effect: Z = 2.87 (P = 0.004)				
**1.8.5 Ecological**				
Too 2020 (mobile plant operators, all genders)	0.1875	0.0734	2.5%	1.21 [1.04, 1.39]
Too 2020 (road and rail drivers, all genders)	0.7303	0.0361	2.6%	2.08 [1.93, 2.23]
**Subtotal (95% CI)**			**5.1%**	**1.59 [0.93, 2.70]**
Heterogeneity: Tau² = 0.14; Chi² = 44.04, df = 1 (P < 0.00001); I² = 98%				
Test for overall effect: Z = 1.70 (P = 0.09)				
**Total (95% CI)**			**100.0%**	**1.32 [1.15, 1.52]**
Heterogeneity: Tau² = 0.20; Chi² = 901.02, df = 52 (P < 0.00001); I² = 94%				
Test for overall effect: Z = 3.83 (P = 0.0001)				
Test for subgroup differences: Chi² = 10.03, df = 4 (P = 0.04), I² = 60.1%				

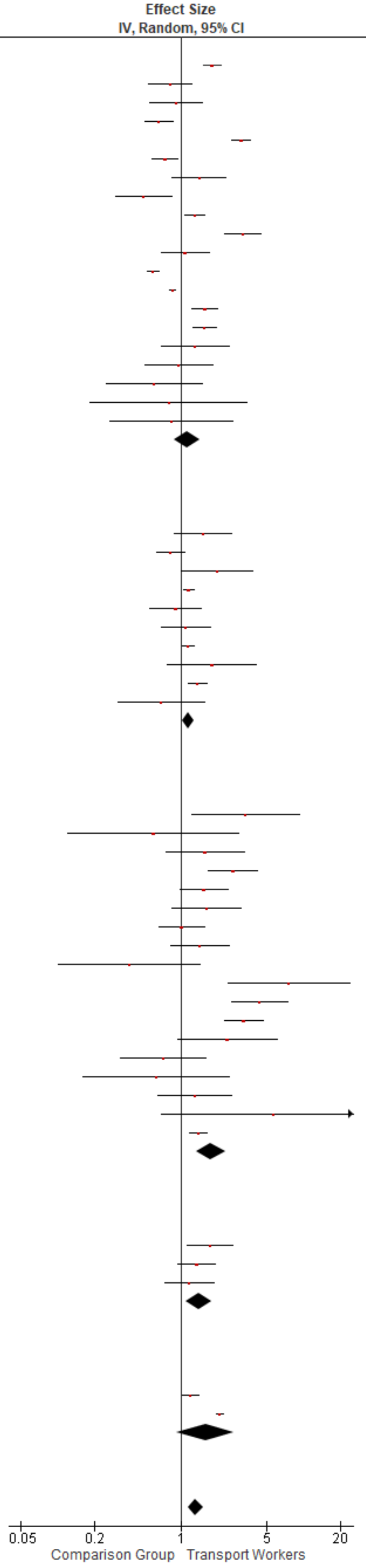

H. By Adjustment

Study or Subgroup	log[Effect Size]	SE	Weight	Effect Size IV, Random, 95% CI
**1.9.1 Age-adjusted**				
Ballard 2002 (cabin attendant, females)	1.2179	0.5116	1.1%	3.38 [1.24, 9.21]
Ballard 2002 (cabin attendant, males)	-0.5108	0.8212	0.6%	0.60 [0.12, 3.00]
Ballard 2002 (cockpit crew, males)	0.4574	0.3734	1.5%	1.58 [0.76, 3.28]
Brandt 1994 (deck crew, 20-34y, males)	0.9821	0.2333	2.1%	2.67 [1.69, 4.22]
Brandt 1994 (deck crew, 35-49y, males)	0.4318	0.2306	2.1%	1.54 [0.98, 2.42]
Brandt 1994 (deck crew, 50-64y, males)	0.4886	0.3322	1.7%	1.63 [0.85, 3.13]
Brandt 1994 (deck officers, 20-34y, males)	0.0198	0.2221	2.1%	1.02 [0.66, 1.58]
Brandt 1994 (deck officers, 35-49y, males)	0.3577	0.2776	1.9%	1.43 [0.83, 2.46]
Brandt 1994 (deck officers, 50-64y, males)	-0.9676	0.6811	0.8%	0.38 [0.10, 1.44]
Brandt 1994 (engine crew, 20-34y, males)	2.0281	0.5818	1.0%	7.60 [2.43, 23.77]
Brandt 1994 (engine crew, 35-49y, males)	1.4816	0.2684	1.9%	4.40 [2.60, 7.45]
Brandt 1994 (engine crew, 50-64y, males)	1.1787	0.1854	2.2%	3.25 [2.26, 4.67]
Brandt 1994 (engine officers, 20-34y, males)	0.8755	0.4782	1.2%	2.40 [0.94, 6.13]
Brandt 1994 (engine officers, 35-49y, males)	-0.3285	0.4137	1.4%	0.72 [0.32, 1.62]
Brandt 1994 (engine officers, 50-64y, males)	-0.462	0.6993	0.8%	0.63 [0.16, 2.48]
Guseva Canu 2019 (garbage collectors, males)	0.4187	0.2731	1.9%	1.52 [0.89, 2.60]
Guseva Canu 2019 (locomotive eng drivers, males)	-0.1863	0.1326	2.4%	0.83 [0.64, 1.08]
Guseva Canu 2019 (motor vehicle drivers, females)	0.6831	0.3384	1.7%	1.98 [1.02, 3.84]
Guseva Canu 2019 (motor vehicle drivers, males)	0.1484	0.0508	2.6%	1.16 [1.05, 1.28]
Guseva Canu 2019 (ship, air control&tech, males)	-0.0943	0.2477	2.0%	0.91 [0.56, 1.48]
Guseva Canu2019(transportclerks,recording,females)	0.0953	0.238	2.0%	1.10 [0.69, 1.75]
Guseva Canu2019(transport clerks,recording, males)	0.1398	0.0562	2.6%	1.15 [1.03, 1.28]
Hansen 1998 (seafarers, females)	1.7334	1.0737	0.4%	5.66 [0.69, 46.42]
Kposowa 1999 (transport equip operatives, all)	0.5878	0.4267	1.4%	1.80 [0.78, 4.15]
Mustard 2010 (motor transport, males)	0.3192	0.0938	2.5%	1.38 [1.14, 1.65]
Mustard 2010 (railway transport, males)	-0.3696	0.4139	1.4%	0.69 [0.31, 1.56]
Rafnsson 1993 (seamen, males)	0.3293	0.0836	2.5%	1.39 [1.18, 1.64]
Stallones 2013 (transport,material moving,females)	-0.5225	0.0571	2.6%	0.59 [0.53, 0.66]
Stallones 2013 (transport, material moving, males)	-0.1507	0.0257	2.6%	0.86 [0.82, 0.90]
Wada 2016 (transport, machine operation, males)	0.4574	0.1236	2.4%	1.58 [1.24, 2.01]
**Subtotal (95% CI)**			**53.4%**	**1.37 [1.16, 1.61]**
Heterogeneity: Tau² = 0.12; Chi² = 308.29, df = 29 (P < 0.00001); I² = 91%				
Test for overall effect: Z = 3.77 (P = 0.0002)				
**1.9.2 Multiple-adjusted**				
Guidotti 1992 (bus drivers, males)	0.2647	0.358	1.6%	1.30 [0.65, 2.63]
Windsor-Shellard 2019 (forklift drivers, males)	0.4511	0.1163	2.4%	1.57 [1.25, 1.97]
**Subtotal (95% CI)**			**4.0%**	**1.54 [1.24, 1.92]**
Heterogeneity: Tau² = 0.00; Chi² = 0.25, df = 1 (P = 0.62); I² = 0%				
Test for overall effect: Z = 3.92 (P < 0.0001)				
**1.9.3 Unadjusted**				
Agerbo 2007 (drivers&mobile plant operators, all)	0.5596	0.2187	2.1%	1.75 [1.14, 2.69]
Agerbo 2007 (motor-vehicle drivers, all genders)	0.3001	0.1793	2.3%	1.35 [0.95, 1.92]
Agerbo 2007 (transport and other labourers, all)	0.1655	0.2312	2.1%	1.18 [0.75, 1.86]
Andersen 2010 (general transport, all genders)	0.5878	0.0863	2.5%	1.80 [1.52, 2.13]
Roberts 2013a(dockers and stevedores, all genders)	-0.1905	0.2092	2.1%	0.83 [0.55, 1.25]
Roberts 2013a (driving instructors, all genders)	-0.0827	0.2505	2.0%	0.92 [0.56, 1.50]
Roberts 2013a(forklift truck drivers, all genders)	-0.4112	0.1371	2.4%	0.66 [0.51, 0.87]
Roberts 2013a (merchant seafarers, all genders)	1.1398	0.0886	2.5%	3.13 [2.63, 3.72]
Roberts 2013a (rail operating staff, all genders)	-0.2893	0.1251	2.4%	0.75 [0.59, 0.96]
Roberts 2013b(dockers and stevedores, all genders)	0.3498	0.2587	2.0%	1.42 [0.85, 2.36]
Roberts 2013b (driving instructors, all genders)	-0.6938	0.2677	1.9%	0.50 [0.30, 0.84]
Roberts 2013b(forklift truck drivers, all genders)	0.2667	0.0954	2.5%	1.31 [1.08, 1.57]
Roberts 2013b (merchant seafarers, all genders)	1.1692	0.1722	2.3%	3.22 [2.30, 4.51]
Roberts 2013b (rail operating staff, all genders)	0.0851	0.23	2.1%	1.09 [0.69, 1.71]
Too 2020 (mobile plant operators, all genders)	0.1875	0.0734	2.5%	1.21 [1.04, 1.39]
Too 2020 (road and rail drivers, all genders)	0.7303	0.0361	2.6%	2.08 [1.93, 2.23]
Yamauchi 2018 (transport&postal, 30-39y, males)	0.2683	0.3272	1.7%	1.31 [0.69, 2.48]
Yamauchi 2018 (transport&postal, 40-49y, males)	-0.0339	0.3258	1.7%	0.97 [0.51, 1.83]
Yamauchi 2018 (transport&postal, 50-59y, males)	-0.499	0.4596	1.3%	0.61 [0.25, 1.49]
Yamauchi 2018 (transport&postal, over 60y, males)	-0.2231	0.7506	0.7%	0.80 [0.18, 3.48]
Yamauchi 2018 (transport&postal, under30y, males)	-0.1744	0.5927	1.0%	0.84 [0.26, 2.68]
**Subtotal (95% CI)**			**42.6%**	**1.23 [0.99, 1.53]**
Heterogeneity: Tau² = 0.20; Chi² = 269.79, df = 20 (P < 0.00001); I² = 93%				
Test for overall effect: Z = 1.90 (P = 0.06)				
**Total (95% CI)**			**100.0%**	**1.32 [1.15, 1.52]**
Heterogeneity: Tau² = 0.20; Chi² = 901.02, df = 52 (P < 0.00001); I² = 94%				
Test for overall effect: Z = 3.83 (P = 0.0001)				
Test for subgroup differences: Chi² = 2.04, df = 2 (P = 0.36), I² = 1.7%				

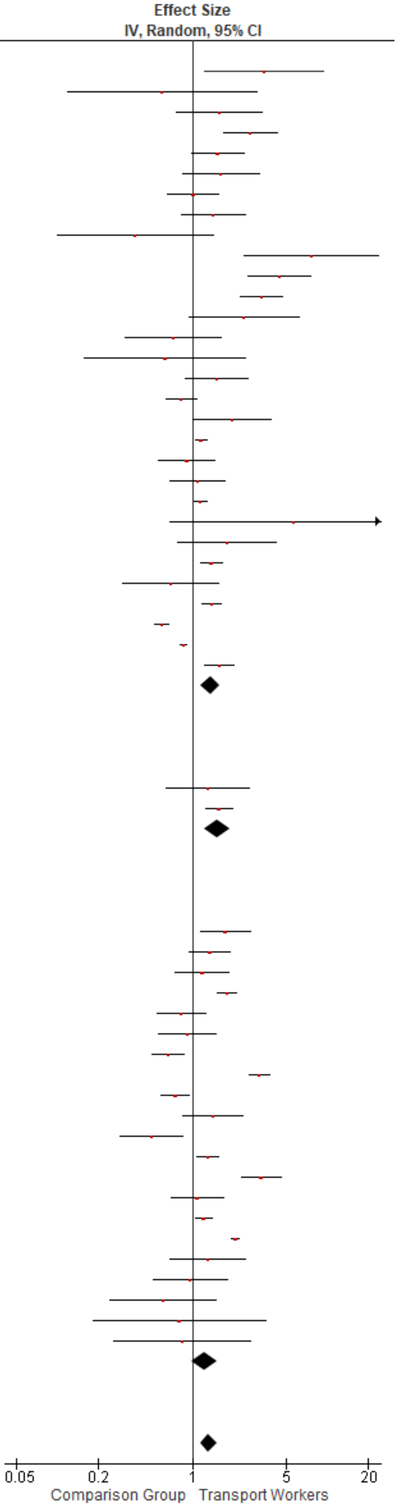

I. By Study Quality

Study or Subgroup	log[Effect Size]	SE	Weight	Effect Size IV, Random, 95% CI
**1.10.1 Low**				
Roberts 2013a(dockers and stevedores, all genders)	-0.1905	0.2092	2.1%	0.83 [0.55, 1.25]
Roberts 2013a (driving instructors, all genders)	-0.0827	0.2505	2.0%	0.92 [0.56, 1.50]
Roberts 2013a(forklift truck drivers, all genders)	-0.4112	0.1371	2.4%	0.66 [0.51, 0.87]
Roberts 2013a (merchant seafarers, all genders)	1.1398	0.0886	2.5%	3.13 [2.63, 3.72]
Roberts 2013a (rail operating staff, all genders)	-0.2893	0.1251	2.4%	0.75 [0.59, 0.96]
Roberts 2013b(dockers and stevedores, all genders)	0.3498	0.2587	2.0%	1.42 [0.85, 2.36]
Roberts 2013b (driving instructors, all genders)	-0.6938	0.2677	1.9%	0.50 [0.30, 0.84]
Roberts 2013b(forklift truck drivers, all genders)	0.2667	0.0954	2.5%	1.31 [1.08, 1.57]
Roberts 2013b (merchant seafarers, all genders)	1.1692	0.1722	2.3%	3.22 [2.30, 4.51]
Roberts 2013b (rail operating staff, all genders)	0.0851	0.23	2.1%	1.09 [0.69, 1.71]
Yamauchi 2018 (transport&postal, 30-39y, males)	0.2683	0.3272	1.7%	1.31 [0.69, 2.48]
Yamauchi 2018 (transport&postal, 40-49y, males)	-0.0339	0.3258	1.7%	0.97 [0.51, 1.83]
Yamauchi 2018 (transport&postal, 50-59y, males)	-0.499	0.4596	1.3%	0.61 [0.25, 1.49]
Yamauchi 2018 (transport&postal, over 60y, males)	-0.2231	0.7506	0.7%	0.80 [0.18, 3.48]
Yamauchi 2018 (transport&postal, under30y, males)	-0.1744	0.5927	1.0%	0.84 [0.26, 2.68]
**Subtotal (95% CI)**			**28.5%**	**1.08 [0.76, 1.55]**
Heterogeneity: Tau² = 0.42; Chi² = 191.93, df = 14 (P < 0.00001); I² = 93%				
Test for overall effect: Z = 0.44 (P = 0.66)				
**1.10.2 Moderate**				
Ballard 2002 (cabin attendant, females)	1.2179	0.5116	1.1%	3.38 [1.24, 9.21]
Ballard 2002 (cabin attendant, males)	-0.5108	0.8212	0.6%	0.60 [0.12, 3.00]
Ballard 2002 (cockpit crew, males)	0.4574	0.3734	1.5%	1.58 [0.76, 3.28]
Guidotti 1992 (bus drivers, males)	0.2647	0.358	1.6%	1.30 [0.65, 2.63]
Hansen 1998 (seafarers, females)	1.7334	1.0737	0.4%	5.66 [0.69, 46.42]
Rafnsson 1993 (seamen, males)	0.3293	0.0836	2.5%	1.39 [1.18, 1.64]
Stallones 2013 (transport,material moving,females)	-0.5225	0.0571	2.6%	0.59 [0.53, 0.66]
Stallones 2013 (transport, material moving, males)	-0.1507	0.0257	2.6%	0.86 [0.82, 0.90]
Wada 2016 (transport, machine operation, males)	0.4574	0.1236	2.4%	1.58 [1.24, 2.01]
**Subtotal (95% CI)**			**15.4%**	**1.18 [0.88, 1.58]**
Heterogeneity: Tau² = 0.12; Chi² = 113.96, df = 8 (P < 0.00001); I² = 93%				
Test for overall effect: Z = 1.09 (P = 0.28)				
**1.10.3 High**				
Agerbo 2007 (drivers&mobile plant operators, all)	0.5596	0.2187	2.1%	1.75 [1.14, 2.69]
Agerbo 2007 (motor-vehicle drivers, all genders)	0.3001	0.1793	2.3%	1.35 [0.95, 1.92]
Agerbo 2007 (transport and other labourers, all)	0.1655	0.2312	2.1%	1.18 [0.75, 1.86]
Andersen 2010 (general transport, all genders)	0.5878	0.0863	2.5%	1.80 [1.52, 2.13]
Brandt 1994 (deck crew, 20-34y, males)	0.9821	0.2333	2.1%	2.67 [1.69, 4.22]
Brandt 1994 (deck crew, 35-49y, males)	0.4318	0.2306	2.1%	1.54 [0.98, 2.42]
Brandt 1994 (deck crew, 50-64y, males)	0.4886	0.3322	1.7%	1.63 [0.85, 3.13]
Brandt 1994 (deck officers, 20-34y, males)	0.0198	0.2221	2.1%	1.02 [0.66, 1.58]
Brandt 1994 (deck officers, 35-49y, males)	0.3577	0.2776	1.9%	1.43 [0.83, 2.46]
Brandt 1994 (deck officers, 50-64y, males)	-0.9676	0.6811	0.8%	0.38 [0.10, 1.44]
Brandt 1994 (engine crew, 20-34y, males)	2.0281	0.5818	1.0%	7.60 [2.43, 23.77]
Brandt 1994 (engine crew, 35-49y, males)	1.4816	0.2684	1.9%	4.40 [2.60, 7.45]
Brandt 1994 (engine crew, 50-64y, males)	1.1787	0.1854	2.2%	3.25 [2.26, 4.67]
Brandt 1994 (engine officers, 20-34y, males)	0.8755	0.4782	1.2%	2.40 [0.94, 6.13]
Brandt 1994 (engine officers, 35-49y, males)	-0.3285	0.4137	1.4%	0.72 [0.32, 1.62]
Brandt 1994 (engine officers, 50-64y, males)	-0.462	0.6993	0.8%	0.63 [0.16, 2.48]
Guseva Canu 2019 (garbage collectors, males)	0.4187	0.2731	1.9%	1.52 [0.89, 2.60]
Guseva Canu 2019 (locomotive eng drivers, males)	-0.1863	0.1326	2.4%	0.83 [0.64, 1.08]
Guseva Canu 2019 (motor vehicle drivers, females)	0.6831	0.3384	1.7%	1.98 [1.02, 3.84]
Guseva Canu 2019 (motor vehicle drivers, males)	0.1484	0.0508	2.6%	1.16 [1.05, 1.28]
Guseva Canu 2019 (ship, air control&tech, males)	-0.0943	0.2477	2.0%	0.91 [0.56, 1.48]
Guseva Canu2019(transportclerks,recording,females)	0.0953	0.238	2.0%	1.10 [0.69, 1.75]
Guseva Canu2019(transport clerks,recording, males)	0.1398	0.0562	2.6%	1.15 [1.03, 1.28]
Kposowa 1999 (transport equip operatives, all)	0.5878	0.4267	1.4%	1.80 [0.78, 4.15]
Mustard 2010 (motor transport, males)	0.3192	0.0938	2.5%	1.38 [1.14, 1.65]
Mustard 2010 (railway transport, males)	-0.3696	0.4139	1.4%	0.69 [0.31, 1.56]
Too 2020 (mobile plant operators, all genders)	0.1875	0.0734	2.5%	1.21 [1.04, 1.39]
Too 2020 (road and rail drivers, all genders)	0.7303	0.0361	2.6%	2.08 [1.93, 2.23]
Windsor-Shellard 2019 (forklift drivers, males)	0.4511	0.1163	2.4%	1.57 [1.25, 1.97]
**Subtotal (95% CI)**			**56.1%**	**1.48 [1.27, 1.71]**
Heterogeneity: Tau² = 0.11; Chi² = 235.00, df = 28 (P < 0.00001); I² = 88%				
Test for overall effect: Z = 5.10 (P < 0.00001)				
**Total (95% CI)**			**100.0%**	**1.32 [1.15, 1.52]**
Heterogeneity: Tau² = 0.20; Chi² = 901.02, df = 52 (P < 0.00001); I² = 94%				
Test for overall effect: Z = 3.83 (P = 0.0001)				
Test for subgroup differences: Chi² = 3.58, df = 2 (P = 0.17), I² = 44.2%				

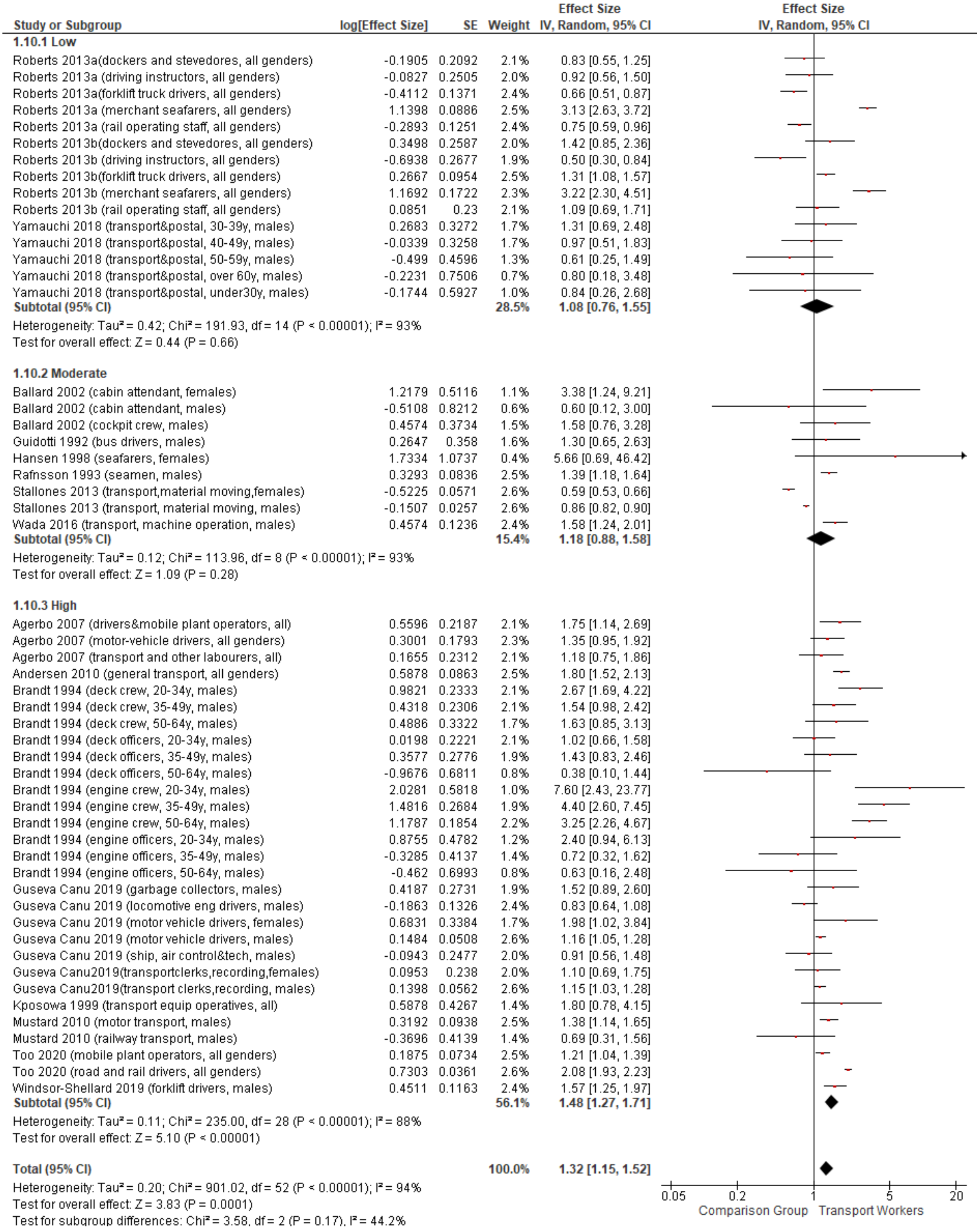

References (Supplementary Materials)

Agerbo, E., Gunnell, D., Bonde, J. P., Mortensen, P. B., & Nordentoft, M. (2007). Suicide and occupation: the impact of socio-economic, demographic and psychiatric differences. *Psychological Medicine*, *37*(8), 1131.

Andersen, K., Hawgood, J., Klieve, H., Kõlves, K., & De Leo, D. (2010). Suicide in selected occupations in Queensland: evidence from the State suicide register. *Australian & New Zealand Journal of Psychiatry*, *44*(3), 243-249.

Ballard, T. J., Lagorio, S., De Santis, M., De Angelis, G., Santaquilani, M., Caldora, M., & Verdecchia, A. (2002). A retrospective cohort mortality study of Italian commercial airline cockpit crew and cabin attendants, 1965–96. *International Journal of Occupational and Environmental Health*, *8*(2), 87-96.

Brandt, L., Kirk, N., Jensen, O., & Hansen, H. (1994). Mortality among Danish merchant seamen from 1970 to 1985. *American Journal of Industrial Medicine*, *25*(6), 867-876.

Guidotti, T. (1992). Mortality of urban transit workers: indications of an excess of deaths by suicide using gas. *Occupational Medicine*, *42*(3), 125-128.

Guseva Canu, I., Bovio, N., Mediouni, Z., Bochud, M., & Wild, P. (2019). Suicide mortality follow-up of the Swiss National Cohort (1990–2014): sex-specific risk estimates by occupational socio-economic group in working-age population. *Social Psychiatry and Psychiatric Epidemiology*, *54*(12), 1483-1495.

Hansen, H. L., & Jensen, J. (1998). Female seafarers adopt the high risk lifestyle of male seafarers. *Occupational and Environmental Medicine*, *55*(1), 49-51.

Kmet, L. M., Lee, R. C., & Cook, L. S. (2004). *Standard quality assessment criteria for evaluating primary research papers from a variety of fields.* Alberta Heritage Foundation for Medical Research, Edmonton, Alberta: Canada

Kposowa, A. J. (1999). Suicide mortality in the United States: differentials by industrial and occupational groups. *American Journal of Industrial Medicine*, *36*(6), 645-652.

Labovitz, S., & Hagedorn, R. (1971). An analysis of suicide rates among occupational categories. *Sociological Inquiry*, *41*(1), 67-72.

Meltzer, H., Griffiths, C., Brock, A., Rooney, C., & Jenkins, R. (2008). Patterns of suicide by occupation in England and Wales: 2001–2005. *The British Journal of Psychiatry*, *193*(1), 73-76.

Milner, A., Page, K., & LaMontagne, A. D. (2015). Suicide among male road and rail drivers in Australia: a retrospective mortality study. *Road & Transport Research: A Journal of Australian and New Zealand Research and Practice*, *24*(2), 26.

Milner, A., Witt, K., Maheen, H., & LaMontagne, A. (2017). Access to means of suicide, occupation and the risk of suicide: a national study over 12 years of coronial data. *BMC Psychiatry*, *17*(1), 1-7.

Mustard, C. A., Bielecky, A., Etches, J., Wilkins, R., Tjepkema, M., Amick, B. C., Smith, P. M., Gnam, W. H., & Aronson, K. J. (2010). Suicide mortality by occupation in Canada, 1991–2001. *The Canadian Journal of Psychiatry*, *55*(6), 369-376.

Page, M. J., McKenzie, J. E., Bossuyt, P. M., Boutron, I., Hoffmann, T. C., Mulrow, C. D., Shamseer, L., Tetzlaff, J. M., Akl, E. A., & Brennan, S. E. (2021). The PRISMA 2020 statement: an updated guideline for reporting systematic reviews. *British Medical Journal*, *372*.

Rafnsson, V., & Gunnarsdottir, H. (1993). Risk of fatal accidents occurring other than at sea among Icelandic seamen. *British Medical Journal*, *306*(6889), 1379-1381.

Reardon, T., Harvey, K., Baranowska, M., O'Brien, D., Smith, L., & Creswell, C. (2017). What do parents perceive are the barriers and facilitators to accessing psychological treatment for mental health problems in children and adolescents? A systematic review of qualitative and quantitative studies. *European Child & Adolescent Psychiatry*, *26*(6), 623-647.

Roberts, S., Jaremin, B., & Lloyd, K. (2013). High-risk occupations for suicide. *Psychological Medicine*, *43*(6), 1231-1240.

Schmid, M., Michaud, L., Bovio, N., & Guseva Canu, I. (2020). Prevalence of somatic and psychiatric morbidity across occupations in Switzerland and its correlation with suicide mortality: results from the Swiss National Cohort (1990–2014). *BMC psychiatry*, *20*(1), 1-13.

Stack, S. (2001). Occupation and suicide. *Social Science Quarterly*, *82*(2), 384-396.

Stallones, L., Doenges, T., Dik, B. J., & Valley, M. A. (2013). Occupation and suicide: Colorado, 2004–2006. *American Journal of Industrial Medicine*, *56*(11), 1290-1295.

Too, L. S., & Spittal, M. J. (2020). Suicide Clusters Among Top 10 High-Risk Occupations: A Study From 2001 to 2016 in Australia. *The Journal of Nervous and Mental Disease*, *208*(12), 942-946.

Wada, K., Eguchi, H., Prieto-Merino, D., & Smith, D. R. (2016). Occupational differences in suicide mortality among Japanese men of working age. *Journal of Affective Disorders*, *190*, 316-321.

Wild, P., Bovio, N., & Guseva Canu, I. (2021). Part-time work and other occupational risk factors for suicide among working women in the Swiss National Cohort. *International Archives of Occupational and Environmental Health*, 1-10.

Windsor-Shellard, B., & Gunnell, D. (2019). Occupation-specific suicide risk in England: 2011–2015. *The British Journal of Psychiatry*, *215*(4), 594-599.

Yamauchi, T., Sasaki, T., Yoshikawa, T., Matsumoto, S., & Takahashi, M. (2018). Incidence of overwork-related mental disorders and suicide in Japan. *Occupational Medicine*, *68*(6), 370-377.
